# The Early Nutrition Paradox in Preterm and Intrauterine Growth Restricted (IUGR) Infants: A Critical Review of the Developmental Programming Hypothesis and the Limits of Its Evidence

**DOI:** 10.3390/nu18213542

**Published:** 2026-10-27

**Authors:** Arieh Riskin, Adir Iofe, Rasha Zoabi Safadi, Naama Tal, Ayala Gover

**Affiliations:** 1 Department of Neonatology, Bnai Zion Medical Center, Haifa 3339419, Israel; adir.iofe@b-zion.org.il (A.I.); rasha.safadi@b-zion.org.il (R.Z.S.); naama.tal@b-zion.org.il (N.T.); ayala.gover@b-zion.org.il (A.G.); 2 Ruth & Bruce Rappaport Faculty of Medicine, Technion Israel Institute of Technology, Haifa 3525433, Israel

**Keywords:** developmental programming, developmental origins of health and disease (DOHaD), prematurity, intrauterine growth restriction (IUGR), infant nutrition, metabolic syndrome

## Abstract

Nutrition during early life may be an important determinant of long-term health. Preterm birth, affecting approximately 10% of deliveries worldwide, and intrauterine growth restriction (IUGR) are major perinatal conditions. The Developmental Origins of Health and Disease (DOHaD) hypothesis proposes that early nutritional and environmental exposures shape adult disease risk. This narrative review synthesizes epidemiological, cohort, animal, and interventional evidence on early nutrition and long-term outcomes in these infants. Rapid weight gain has been associated with later obesity and cardio-metabolic risk; however, this does not show that the tempo of gain, rather than attained size, carries the risk, and catch-up growth after preterm birth or growth restriction supports neurodevelopment. Preterm birth has been associated with a 53% higher risk of ischemic heart disease, whereas IUGR confers overlapping but distinct risks. Hypothesized mechanisms include epigenetic modification, structural organ deficits, hormonal reprogramming, gut-microbiome dysbiosis, and oxidative stress. Because the human evidence is almost entirely observational and vulnerable to confounding and to over-adjustment for attained body size, these associations should be read as hypotheses rather than established causal effects. Individualized, guideline-based and responsive feeding strategies, with support for mothers to provide their own milk, are needed to optimize immediate outcomes while limiting lifelong disease susceptibility.

## 1. Introduction

The relationship between early nutrition and long-term health has emerged as one of the most significant paradigms in modern medicine [1]. The focus of nutritional research has shifted fundamentally from simply meeting basic nutritional requirements to understanding how early nutritional experiences can permanently alter physiological function and disease susceptibility throughout life, a concept referred to as nutritional programming [2,3]. This concept falls within developmental programming, also known as the Developmental Origins of Health and Disease (DOHaD). DOHaD is a hypothesis rather than an established causal framework; it proposes that events in fetal and early postnatal life have consequences that extend across the entire lifespan [4,5,6].

Preterm birth, defined as delivery before 37 completed weeks of gestation, affects approximately 10.6% of all live births globally, representing over 15 million infants annually, and is the leading cause of neonatal mortality and morbidity worldwide [7]. Intrauterine growth restriction (IUGR) is defined as a fetal growth rate below the expected growth potential, whereas small for gestational age (SGA) is defined as birth weight below the 10th percentile for gestational age, regardless of growth trajectory during pregnancy. Infants born preterm, and those with IUGR, face not only the immediate challenges of prematurity but also a lifelong legacy of increased vulnerability to chronic non-communicable diseases [8,9,10].

Evidence from epidemiological studies, animal models and experimental interventions is consistent with the hypothesis that in utero exposures, prematurity and postnatal experiences have long-term health implications, although the human evidence is largely observational (Section 4.5). Studies on nutrition and growth suggest that inadequate growth and nutrition during critical developmental periods may have lasting adverse consequences. Conversely, it has been hypothesized that ‘accelerated’ or excessively rapid infant growth increases the propensity to components of the metabolic syndrome, including glucose intolerance, obesity, elevated blood pressure and dyslipidemia, which collectively predispose to cardiovascular morbidity and mortality in adulthood [11]. Rapid weight gain in infancy is consistently associated with later obesity [12]; however, because a child who is heavier at a given age must have gained weight faster to reach that size, these associations do not by themselves show that the tempo of gain, rather than attained size, carries the risk, nor that gain in infancy matters more than gain later in childhood or adolescence. In infants born preterm or growth-restricted, catch-up growth is, moreover, regarded as a desirable recovery [13]. For preterm infants, this creates a unique and complex clinical challenge [8]. Adequate nutrition is essential for immediate survival, growth, and neurodevelopmental outcomes in the postnatal period [8,14,15,16]; yet the long-term metabolic consequences of nutritional strategies must also be carefully considered [17,18]. Similarly, infants with IUGR face a complex interplay between the potential benefits of postnatal catch-up growth and the programming risks associated with accelerated weight gain [19,20].

This review examines the current understanding of how early nutrition may influence long-term health outcomes. It explores the hypothesized mechanisms of developmental programming, including the increasingly recognized roles of epigenetics, the gut microbiome, and oxidative stress; reviews the clinical evidence for long-term cardiovascular and metabolic consequences in preterm and IUGR populations; and discusses clinical implications for the nutritional management of these vulnerable infants. The preconception, prenatal and early-childhood periods are included deliberately and are treated only insofar as they bear on the preterm and IUGR infant: the preconception and prenatal windows determine whether an infant is born preterm or growth-restricted at all and set the baseline against which postnatal nutrition acts, while the early-childhood period is where the growth trajectory established in the neonatal unit is either consolidated or corrected. Sections addressing these adjacent periods have been kept deliberately brief relative to the neonatal sections.

### Scope, Literature Search and Appraisal of the Evidence

This is a narrative rather than a systematic review, and it does not claim the reproducibility of a systematic search. To improve transparency, we describe the approach taken. PubMed/MEDLINE, Embase and the Cochrane Library were searched from inception to March 2026, supplemented by hand-searching of reference lists of retrieved articles and of the websites of ESPGHAN, ESPEN, the WHO and the American Academy of Pediatrics. Searches were restricted to English-language publications. Search terms combined the population (“preterm”, “premature”, “very low birth weight”, “intrauterine growth restriction”, “fetal growth restriction”, “small for gestational age”) with the exposure (“nutrition”, “protein intake”, “human milk”, “breastfeeding”, “fortification”, “catch-up growth”, “postnatal growth”) and with the outcome or mechanism (“developmental programming”, “DOHaD”, “metabolic syndrome”, “blood pressure”, “insulin resistance”, “obesity”, “cardiovascular”, “epigenetic”, “DNA methylation”, “microbiome”, “oxidative stress”, “body composition”).

Where sources conflicted, evidence was prioritized in the following order: (i) current clinical practice guidelines and society position papers; (ii) systematic reviews and meta-analyses and individual-participant-data meta-analyses in particular; (iii) randomized controlled trials with long-term follow-up; (iv) large population-based or national-registry cohorts with sibling or within-family comparisons; (v) smaller observational cohorts and cross-sectional studies; and (vi) animal and in-vitro mechanistic work. Categories (v) and (vi) are used to generate hypotheses about mechanism and are identified as such in the text; they are not used to support clinical recommendations.

A caveat applies to the whole of this literature and is developed in Section 4.5. Randomizing pregnant women or their infants to under- or over-nutrition is not ethically possible, so with the partial exception of neonatal dietary trials the human evidence for developmental programming is necessarily observational. It is therefore open to confounding—by social determinants of health, maternal adiposity, maternal hypertensive disease and neonatal morbidity—and, in a large proportion of published analyses, to over-adjustment for body size attained after the exposure. Readers should treat the associations described below as hypotheses supported to varying degrees, not as demonstrated causal effects.

Supplementary Figure S1 is a schematic of the DOHaD hypothesis across the life course. It depicts the proposed critical windows for exposure, the proposed mechanisms, and the divergent preterm and IUGR adult phenotypes reported in a single cohort. It represents the hypothesis as it has been formulated, not a set of demonstrated causal pathways; the strength of the evidence for each element is discussed in Section 3 and Section 4.5. It does not show the maternal and social factors that may confound the associations it depicts (Section 4.5), and it should not be read as evidence for the pathways it illustrates. For this reason, it is provided in the Supplementary Material rather than in the main text.

## 2. Historical Perspective and Theoretical Basis

### 2.1. Barker’s Hypothesis and Fetal Programming

The foundation of our current understanding of developmental programming can be traced to the pioneering work of David Barker in the late 1980s. Barker’s seminal ecological observations showed a strong geographical correlation between areas of high infant mortality (a marker of poverty) in early 20th-century England and subsequent high rates of coronary artery disease mortality 50–70 years later [21]. This striking finding challenged the prevailing assumption that coronary disease was a disease of affluence caused by a high-fat diet and a sedentary lifestyle, and led to the formulation of “Barker’s hypothesis” or the “fetal origins of adult disease” concept, which proposed that conditions in utero during embryonic and fetal life could influence the development of tissues and organs in ways that alter lifelong disease risk [6,22].

Barker hypothesized that small size at birth, reflecting poor fetal nutrition, has a causal relationship with the development of hypertension, coronary heart disease, and non-insulin-dependent diabetes in middle age [23]. Importantly, the low birth weight (LBW) infants in his early studies were predominantly small for gestational age (SGA) and IUGR cases, as the survival rate of appropriate for gestational age extreme premature infants was very low during the time periods examined [24]. Gestational age was not reliably recorded in these historical records, so the exposure in Barker’s analyses is birth weight rather than growth restriction as such, and the two cannot be separated within that data set (Table 1). Later syntheses have questioned the strength of the central association: in a systematic review of 55 studies, Huxley et al. found that the inverse association between birth weight and later blood pressure became progressively weaker as study size increased and was small in the largest studies [25].

“Programming” is defined as the effect of an endogenous or environmental stimulus or insult that acts at a critical time during development and results in a change in structure or function of an organism or system [1,22,26]. The concept of “fetal programming” proposes that although these physiological adaptations occur in response to transient phenomena such as fetal undernutrition, the changes become permanent because they occur during critical periods of development when organ systems are particularly plastic and susceptible to environmental influences [26,27]. Over the subsequent decades, the field evolved from the narrow “fetal origins” concept to the broader DOHaD hypothesis, encompassing prenatal and early postnatal life, overnutrition as well as undernutrition, and a wide spectrum of adult chronic diseases [22].

### 2.2. The Thrifty Phenotype Hypothesis

Complementing Barker’s earlier observations, Hales and Barker proposed the “thrifty phenotype” hypothesis, which suggests that the fetus responds to nutritional deprivation by developing a metabolic profile that maximizes survival under conditions of limited nutrient availability [28]. This adaptive response involves preferential allocation of limited nutrients to essential organs such as the brain and heart, while reducing the development of other organs including the liver, kidneys, and pancreas.

While this adaptation may improve immediate survival prospects, it creates a “mismatch” when the individual subsequently encounters an environment of nutritional abundance. The thrifty phenotype, optimized for nutrient scarcity, becomes maladaptive in modern environments characterized by caloric excess, leading to increased susceptibility to metabolic disorders [6,29,30]. This concept of “developmental mismatch” has been expanded by Gluckman and Hanson, who proposed that the developing organism uses signals from the environment to construct a phenotype that best matches the predicted postnatal environment; when the actual environment differs from the predicted one, disease risk increases [4].

### 2.3. The Concept of Nutritional Programming

Nutritional programming is a subset of developmental programming, defined as the effect of a nutritional stimulus or insult, which acts at a critical time during development to permanently change the structure and function of an organism or system [31,32]. This concept encompasses several key principles: (1) critical developmental windows when organisms are particularly susceptible to environmental influences; (2) permanent effects that persist throughout the lifespan; (3) dose–response relationships representing graded responses to varying degrees of nutritional stress; and (4) transgenerational effects where programming consequences can be transmitted to subsequent generations.

Animal models have provided experimental support for nutritional programming, although extrapolation from rodent physiology to humans is uncertain. In a mouse model of LBW-associated diabetes, energy restriction during early life, with prevention of early catch-up growth, prevented the development of glucose intolerance and obesity and increased lifespan, while overfeeding led to larger body size, greater internal fat deposition and a considerably shortened lifespan [33,34]. In humans, epidemiological studies have reported associations between low birth weight and adverse long-term health outcomes, and rapid postnatal growth (upward centile crossing) has been proposed to account for part of these associations [35,36]. Lucas coined the term “programming” in the context of early infant nutrition and demonstrated in randomized controlled trials that diet in early postnatal life influences long-term development in preterm infants [26,32].

#### Transgenerational Programming

One of the most striking aspects of developmental programming is its potential to be transmitted across generations. Evidence from animal models shows that nutritional perturbations in pregnancy can affect not only the offspring (F1 generation) but also grandchildren (F2) and potentially great-grandchildren (F3) through both maternal and paternal lineages [37,38,39,40,41,42]. In sheep models, overfeeding during pregnancy results in offspring and grand-offspring who are heavier and show insulin resistance and disrupted leptin signaling, even when the intermediate generation is fed normally. Similarly, fathers fed a high-fat diet in rodent models have daughters (F2) that are larger than controls. In humans, paternal pre-pubertal smoking before age 11 is associated with greater BMI in sons, which has been interpreted as possible epigenetic transmission through the germline [43]. If confirmed in humans, such transgenerational effects would extend the public health implications of suboptimal early nutrition well beyond the individual.

## 3. Hypothesized Mechanisms of Developmental Programming

The mechanisms set out in this section are hypotheses of differing evidential strength, and the section should be read with that in mind. Structural deficits such as reduced nephron endowment rest on direct human autopsy and imaging data and are the best supported. Epigenetic and hormonal mechanisms rest largely on animal models, on cross-sectional human biomarker studies, and on a small number of natural experiments; associations between an early exposure and a later methylation mark do not by themselves establish that the mark mediates the clinical outcome. Microbiome and oxidative-stress mechanisms are supported mainly by animal work and by short-term human studies with surrogate endpoints. No mechanism described below has been shown in humans to carry a causal effect of early nutrition on adult cardio-metabolic disease, and none should be read as an established pathway.

### 3.1. Structural and Functional Organ Development

One of the primary mechanisms through which early experiences and exposures influence long-term health is its effect on organ development during critical periods. The concept of “structural deficits” proposes that inadequate nutrition during organ development leads to reduced numbers of functional units within organs, with lasting consequences for physiological function [44,45,46].

#### 3.1.1. Kidney Development and Hypertension Risk

The relationship between prematurity, kidney development and long-term hypertension risk is the most frequently cited example of developmental programming, and the one with the most direct human data. In humans, nephrogenesis reaches completion at 34–36 weeks of gestation, with more than 60% of nephrons formed during the third trimester [47]. Consequently, nephron endowment is significantly reduced in infants born preterm—potentially by as much as one-quarter to one-third compared to term-born infants.

The hyperfiltration theory hypothesizes that early loss of nephron mass results in compensatory hyperfiltration of remaining nephrons, leading to proteinuria, progressive kidney injury, and increased risk of hypertension in later life [48,49]. This mechanism offers a plausible biological link between preterm birth, reduced nephron number and cardiovascular risk in adulthood. Consistent with it, preterm-born adults have on average higher systolic and diastolic blood pressures than term-born controls [50], although this association does not by itself establish that reduced nephron number is the mediating pathway.

#### 3.1.2. Pancreatic Development and Diabetes Risk

Pancreatic development during fetal life is crucial for long-term glucose homeostasis. In rodent models, maternal protein restriction reduces pancreatic islet cell number and insulin secretory capacity in the offspring [51,52]; it has been proposed that preterm birth has comparable effects in humans and that these contribute to glucose intolerance and diabetes risk in adulthood. The timing of nutritional stress is critical, as pancreatic beta-cell development occurs primarily during the third trimester of pregnancy and early postnatal period. Preterm infants who require parenteral nutrition in early life face periods of relative hyperglycemia and hyperinsulinism that may further affect beta-cell programming.

#### 3.1.3. Other Organ Systems

Programming effects have been proposed for several other organ systems. Reduced muscle mass may lower basal metabolic rate and exercise capacity, predisposing to obesity and metabolic syndrome. Cardiac development in preterm infants involves fewer cardiomyocytes and altered cardiac geometry, which may increase the risk of heart failure and arrhythmias in later life. Altered hepatic development has been proposed to affect lipid metabolism, consistent with recent cohort data in which preterm birth was associated with a 26% higher risk of non-alcoholic fatty liver disease (RR 1.26) [53]. Reduced fat cell progenitor pools, specified during late fetal life, may alter the trajectory of adipose expansion in early postnatal life [54].

### 3.2. Epigenetic Mechanisms

Advances in epigenetics have suggested candidate molecular mechanisms for developmental programming. Epigenetic modifications, including DNA methylation, histone modifications and non-coding RNA regulation, can durably alter gene expression without changing the underlying DNA sequence [40,42,55,56]. Many are established during early development and are mitotically heritable, which offers a possible molecular basis for the persistence of programming effects [8,57].

#### 3.2.1. DNA Methylation

DNA methylation—the addition of a methyl group to cytosine residues at CpG dinucleotides—is the most extensively studied epigenetic modification in developmental programming. Methylation of CpG islands in gene promoter regions generally suppresses transcription, while demethylation permits gene expression. In animal models, diet alters DNA methylation patterns: maternal protein-deficient diets in rats cause DNA hypomethylation at the promoters of several metabolic genes in the offspring [58,59]. The agouti mouse model illustrates how epigenetic modifications can influence both phenotype and disease susceptibility: hypermethylation of the agouti gene promoter, promoted by dietary methyl donors including folate, vitamin B12, and choline, protects offspring from obesity and its metabolic consequences [60].

The Dutch Hunger Winter cohort provides the most widely cited human evidence for persistent epigenetic differences following early nutritional deprivation. Individuals prenatally exposed to famine during the earliest stages of gestation show approximately 5% less DNA methylation at the IGF-2 gene—measurable 60 years later compared to unexposed same-sex siblings [61,62,63,64]. This differential methylation was specific to early-gestation exposure and not seen in those exposed only in late gestation, consistent with a critical window in early gestation. Whether such methylation differences mediate any clinical outcome has not been established.

#### 3.2.2. Histone Modifications and Chromatin Remodeling

Beyond DNA methylation, histone modifications constitute a second major layer of epigenetic regulation. Post-translational modifications to histone tails—including acetylation, methylation, phosphorylation, and ubiquitination—alter chromatin accessibility and gene transcription. Early nutritional experiences can alter histone modification patterns in a gene-specific manner. For example, in a bovine model, maternal nutrient restriction altered H3K4me3 (active) marks at the promoters of fetal hepatic genes controlling glucose metabolism, adipogenesis and inflammatory pathways [65]. These histone marks are influenced by the availability of nutrition-derived metabolic cofactors, including acetyl-CoA and S-adenosylmethionine (SAMe). For example, SAMe acts as the primary methyl donor required for enzymes to deposit these tags. It has therefore been proposed that fluctuations in these nutrient-dependent cofactors alter the epigenetic programming of key rate-limiting enzymes and transcription factors that drive glucose metabolism, adipogenesis and inflammation [66].

#### 3.2.3. Non-Coding RNAs

Non-coding RNAs—including microRNAs (miRNAs) and long non-coding RNAs (lncRNAs)—constitute an additional regulatory layer linking early nutrition to developmental programming. MicroRNAs post-transcriptionally regulate gene expression by binding to target mRNAs and promoting their degradation or translational repression. Maternal nutritional status influences miRNA expression in the placenta and fetal tissues, with consequences for signaling pathways regulating growth, metabolism, and organ development. Specific miRNAs targeting insulin signaling, adipogenesis, and inflammatory pathways are differentially expressed in growth-restricted fetuses [67].

#### 3.2.4. Epigenetic Clocks and Biological Aging in Preterm Infants

A significant advance in epigenetic research has been the development of “epigenetic clocks”—algorithms that estimate biological age based on patterns of DNA methylation at specific CpG sites. These clocks reveal that biological aging can be accelerated or decelerated relative to chronological age, with potential consequences for long-term health. In the context of preterm birth, the pediatric buccal epigenetic (PedBE) clock has been applied to characterize biological aging in very preterm neonates.

In a prospective cohort study by Gomaa et al. [68], 35 very preterm neonates (24–32 weeks gestation) underwent repeated PedBE clock assessments and brain MRI. Extremely preterm neonates (<28 weeks) showed significantly accelerated epigenetic aging compared to less premature peers. Critically, this accelerated PedBE age at term-equivalent age was independently associated with smaller total cerebral volumes, slower brain growth during the NICU period, and worse cognitive and language scores at 18 months. These findings suggest that epigenetic aging provides a biomarker of neurodevelopmental risk that captures information beyond gestational age alone.

Similarly, pregnancy complications including preeclampsia and gestational diabetes are associated with altered epigenetic gestational age in newborns, potentially mediating their known adverse effects on offspring health [69]. Preeclampsia specifically has been linked to epigenetic modifications in placental trophoblast function, with downstream effects on fetal growth and offspring cardiovascular risk in adulthood [70]. These findings highlight how the epigenetic impact of adverse perinatal conditions begins before birth and can be detected in the newborn period.

### 3.3. Hormonal Programming

It has been proposed, largely on the basis of animal studies, that early nutrition durably affects the hormonal axes that regulate body weight, food intake and metabolism [71,72]. Two main mechanisms have been proposed. First, satiety-signaling alterations: early nutritional experiences may establish higher thresholds for satiety signals, leading to increased food intake throughout life. Second, metabolic set-point changes: permanent alterations in systems controlling energy expenditure and storage. In rodent models, early overnutrition programs hypothalamic circuits involving leptin, insulin, glucagon-like peptide-1 (GLP-1) and neuropeptide Y/AGRP neurons in the arcuate nucleus, leading to increased appetite and reduced energy expenditure [73]. Evidence from individuals exposed to famine prenatally shows persistent epigenetic changes decades later, including altered DNA methylation of the IGF-2 gene [61,62], and altered levels of circulating hormones including leptin and adiponectin that regulate body weight and insulin sensitivity.

### 3.4. The Gut Microbiome in Developmental Programming

The gut microbiome has been proposed as a mediator of developmental programming. The neonatal period represents a critical window for microbiome establishment, with colonization patterns having long-term implications for metabolic health, immune development, and brain function. The “gut–brain axis” and “gut–liver axis” provide direct pathways through which microbiome composition influences systemic metabolic programming [74,75].

Preterm infants are particularly vulnerable to microbiome dysbiosis, due to a number of factors, including delivery by Caesarean section (in many cases), exposure to broad-spectrum antibiotics, prolonged parenteral nutrition, and delayed enteral feeding [8]. The preterm gut microbiome is characterized by reduced diversity, dominance of Enterobacteriaceae, and underrepresentation of beneficial Bifidobacterium and Lactobacillus species. This dysbiosis is associated with increased intestinal permeability (“leaky gut”), systemic inflammation, and altered short-chain fatty acid (SCFA) production.

SCFAs—particularly butyrate, propionate, and acetate—produced by microbial fermentation of dietary fiber play critical roles in intestinal epithelial integrity, immune modulation, and metabolic regulation through activation of G-protein coupled receptors (GPR41, GPR43) on adipose, gut, and immune cells. Altered SCFA production in preterm infants may contribute to adipose tissue programming and insulin resistance in later life [76]. Animal models demonstrate that gut-microbiota-targeted interventions including probiotics, prebiotics, and postbiotics can mitigate developmental programming of metabolic syndrome [77]. Human milk, with its rich content of human milk oligosaccharides (HMOs), plays a key role in shaping a healthy microbiome, promoting Bifidobacterium colonization and SCFA production, which offers a possible mechanism for some of the long-term associations of breastfeeding discussed in Section 6.

### 3.5. Oxidative Stress

Oxidative stress—an imbalance between reactive oxygen species (ROS) production and antioxidant defenses—represents an additional mechanism linking prematurity, early nutrition and programming with long-term cardio-metabolic risk [78,79]. It has been proposed that oxidative stress, by damaging macromolecules and altering enzyme function, impairs the nitric oxide (NO) signaling system, a central regulator of cardiovascular, renal and metabolic homeostasis. In animal models, perturbation of the fetal NO–asymmetric dimethyl-arginine (ADMA) axis is followed by hypertension and chronic kidney disease in adult offspring [80,81]. In preterm infants, oxidative stress is amplified by antioxidant immaturity, supplemental oxygen exposure, infection, and ischemia–reperfusion injury [82]. Maternal obesity and preeclampsia further increase fetal oxidative stress, contributing to programming of the offspring’s cardiovascular system [79]. Antioxidant supplementation—including vitamin E, vitamin C, and N-acetylcysteine—has shown promise in animal models for reprogramming oxidative-stress-mediated metabolic disease, though human evidence remains limited [83].

Table 2 summarizes the main mechanisms of developmental programming associating trigger to molecular or structural change that lead to long-term consequence. These include structural, epigenetic, hormonal, microbiome and oxidative-stress mechanisms.

## 4. Clinical Evidence and Epidemiological Studies by Developmental Programming Windows

Table 3 summarizes selected human studies linking early-life exposures to long-term cardio-metabolic outcomes, chosen because they are the most frequently cited in this literature; they are discussed in the following sub-sections, and the limitations of each are appraised in the fourth column.

### 4.1. Preconception Health: The Critical Period Before Conception

The DOHaD hypothesis has traditionally focused on the fetal and early postnatal periods. However, accumulating evidence demonstrates that the health and nutritional status of both parents before conception—the “preconception period”—exerts a profound independent influence on offspring health [92]. The Lancet Preconception Health Series (2018) highlighted that a woman who is healthy at the time of conception is more likely to have a successful pregnancy and a healthy child, with consequences that can extend across generations.

Poor maternal nutrition and obesity before pregnancy are highly prevalent among women of reproductive age globally, and typical diets fall far short of nutritional recommendations in both developed and developing countries [92]. Key preconception nutritional determinants of offspring health include:

Maternal BMI: Both low BMI (predisposing to IUGR) and obesity (associated with gestational diabetes, macrosomia, and offspring metabolic programming) should be optimized before conception. Maternal obesity programs adipocyte progenitor pools in the fetus, accelerating adipose expansion in early postnatal life and predisposing to childhood and adult obesity [54].

Micronutrient status: Periconceptional folic acid supplementation is well established for neural tube defect prevention. However, its role extends to epigenetic programming—folate is a methyl donor that influences global DNA methylation patterns in the developing embryo. Similarly, vitamin B12, choline, zinc, iron, and iodine status at conception influence fetal brain development and programming [92].

Glycemic control: Women with pre-existing diabetes or insulin resistance should achieve optimal glycemic control before conception. Maternal hyperglycemia programs fetal beta-cell function and adiposity through mechanisms including fetal hyperinsulinism, oxidative stress, secondary hypoxic stress, and epigenetic modification [93].

Ultra-processed food consumption: Recent evidence from a large prospective study (Wang et al. BMJ 2022) [94] found that higher maternal consumption of ultra-processed foods during pregnancy was associated with an excess risk of pediatric obesity in offspring. While the preconception evidence is still emerging, current dietary patterns characterized by high ultra-processed food intake are associated with micronutrient deficiencies, excess calorie intake, and altered gut microbiome—all of which may adversely affect epigenetic programming at or near conception.

Paternal health: The emerging field of “paternal programming” recognizes that paternal diet, BMI, and lifestyle before conception influence offspring health through epigenetic modifications in sperm, including DNA methylation, histone modifications, and small non-coding RNAs [95]. Paternal obesity has been associated with altered metabolic outcomes in offspring in both animal models and human studies.

The preconception period thus represents a critical and largely underutilized opportunity for intervention to optimize developmental programming outcomes. Healthcare professionals caring for women and men of reproductive age should incorporate preconception nutritional assessment and counseling as a standard component of preventive care.

### 4.2. In Utero Growth and Nutrition

#### 4.2.1. Undernutrition During Pregnancy: Historical Famines and Natural Experiments

The Dutch Hunger Winter of 1944–1945 provided a landmark natural experiment in human nutritional programming. During this period, food supplies were severely restricted in western Holland, with daily rations falling to 400–800 kcal/person—less than a quarter of recommended adult intake. Pregnant women and their offspring provided epidemiologists with a unique opportunity to study the effects of precisely timed malnutrition [63,64].

Studies of individuals exposed to famine in utero revealed time-dependent programming effects: early-gestation exposure led to higher adult BMI, elevated cholesterol, and increased coronary heart disease risk—a phenotype consistent with the thrifty phenotype hypothesis. Individuals exposed to famine in late gestation had a different phenotype, characterized primarily by impaired glucose tolerance. These time-dependent effects were among the first demonstrations in humans of critical developmental windows for metabolic programming [63,64]. Long-term epigenetic consequences—measurable reduced IGF-2 gene methylation six decades later—confirmed the biological durability of these programming effects [61,62].

Similar natural experiments have been studied in other populations. The Chinese famine of 1959–1961, the Siege of Leningrad (1941–1944), and seasonal famines in The Gambia have all yielded consistent findings: prenatal undernutrition, particularly in early-to-mid gestation, programs increased adult cardio-metabolic risk. The Gambian studies additionally demonstrate that maternal micronutrient status at the time of conception—influenced by season—alters DNA methylation patterns in offspring, connecting preconception nutrition directly to epigenetic programming [92,96,97].

#### 4.2.2. Asymmetric IUGR: A Particularly High-Risk Phenotype

Asymmetric IUGR, characterized by disproportionate reduction in abdominal circumference while relatively sparing head circumference, represents a particularly high-risk programming phenotype [98,99]. This pattern reflects the fetal response to placental insufficiency, with preferential blood flow to the brain and heart at the expense of abdominal organs. Asymmetric IUGR is primarily a protective fetal adaptation to placental insufficiency. By redistributing blood flow to prioritize the brain and heart, the fetus compromises abdominal organ development. This structural “programming” significantly elevates the risk for lifelong metabolic and cardiovascular diseases [100]. In response to chronic hypoxia and nutrient deprivation, the fetus undergoes hemodynamic redistribution with peripheral vasoconstriction, prioritizing the cerebrovascular and coronary circulations while restricting blood supply to the viscera. Preferential shunting preserves bi-parietal diameter and head circumference—the head-sparing phenomenon. Conversely, abdominal circumference shows a disproportionate reduction, largely driven by depleted glycogen and decreased cell size in the liver, kidneys, and skeletal muscle. The resulting infant has a low weight-to-height ratio, relatively spared cardiac, cerebral, and adrenal development, but disproportionate reduction in kidney, liver, pancreas, and skeletal muscle mass [98,100]. This pattern of organ-specific growth restriction is a powerful predictor for later cardiovascular disease [101]. This is mediated via two main mechanisms—vascular remodeling and metabolic “programming”. Prolonged fetal vasoconstriction and altered hemodynamics lead to permanent structural changes in the large arteries, including increased wall stiffness and greater aortic intima thickness. Reduced mass in key metabolic organs (e.g., pancreas, liver, skeletal muscle) alters fetal endocrine development, predisposing the individual to adult-onset insulin resistance, dyslipidemia, hypertension, and coronary heart disease [100]. Crucially, this vascular remodeling extends to the central nervous system; while the ‘brain-sparing’ phenomenon prioritizes cerebral perfusion, the chronic hemodynamic stress alters cerebrovascular development and impairs autoregulation [102]. This permanent alteration in brain vessel structure, coupled with sustained neuroinflammation and tissue hypoxia, significantly elevates the risk for perinatal brain injury and long-term neurodevelopmental deficits, including cerebral palsy [102,103]. Consequently, what initiates as an adaptive fetal response ultimately functions as a form of adverse neurological programming with lifelong consequences [103].

Preeclampsia is the most common cause of asymmetric IUGR, and the epigenetic mechanisms underlying preeclampsia-related fetal programming are increasingly understood. Ashraf et al. [70] reviewed how epigenetic processes—including aberrant DNA methylation of trophoblast invasion genes, altered histone modifications affecting spiral artery remodeling, and dysregulated miRNA expression—contribute to both the pathogenesis of preeclampsia and the long-term cardiovascular risk imprinted in offspring. This connection between maternal hypertensive disease, placental epigenetic dysfunction, and offspring programming represents an important and underappreciated pathway within the DOHaD hypothesis.

#### 4.2.3. In Utero Overnutrition

In contrast to the extensive epidemiological literature on undernutrition, direct clinical outcome data specifically addressing in utero overnutrition remain comparatively limited within the neonatal and preterm literature. Available evidence is largely mechanistic: maternal obesity and gestational diabetes are associated with programming of hypothalamic appetite-regulating circuits (Section 3.3) and with increased fetal oxidative stress (Section 3.5), both plausible pathways to later obesity and cardio-metabolic disease in offspring. Dedicated cohort studies quantifying long-term outcomes in the offspring of obese mothers, analogous to those available for famine-exposed and preterm-born populations, represent an important gap for future research.

### 4.3. Preterm Birth–Prematurity-Related Long-Term Cardiovascular and Metabolic Outcomes

Preterm birth represents a distinct and increasingly recognized risk factor for adult cardio-metabolic disease, separate from the traditional Barker hypothesis which was primarily based on term-born SGA infants. In contrast to the primarily nutritional programming seen in IUGR, preterm birth is characterized by the arrested or abnormal development of structural organ systems, leading to permanent functional deficits in tissues such as the kidneys and lungs. The growing cohort of preterm survivors—now reaching middle age—has enabled large-scale longitudinal studies of long-term outcomes [32,104].

#### 4.3.1. Ischemic Heart Disease

A large Swedish national cohort study by Crump et al. [9], encompassing 2,141,709 singletons born between 1973 and 1994, demonstrated that gestational age at birth was inversely associated with risk of ischemic heart disease (IHD) in adulthood. At ages 30–43 years, preterm birth (<37 weeks) was associated with a 53% increased risk of IHD (adjusted hazard ratio (HR) 1.53; 95% CI, 1.20–1.94), while early-term birth (37–38 weeks) carried a 19% increased risk (HR 1.19; 95% CI, 1.01–1.40), compared to full-term birth. Importantly, preterm-born women, despite having lower absolute IHD incidence than men, had a proportionally higher relative risk (HR 1.93 vs. 1.37 for men). These associations were not explained by shared familial (genetic or environmental) factors in co-sibling analyses, supporting a causal role for preterm birth itself [9].

#### 4.3.2. Metabolic Syndrome Components

A systematic review and meta-analysis by Markopoulou et al. [50], encompassing 43 studies with 18,295 preterm and 294,063 term-born adults, demonstrated that prematurity is significantly associated with multiple components of the metabolic syndrome. Preterm-born adults had significantly higher fat mass (*p* = 0.03), systolic blood pressure (*p* < 0.0001), diastolic blood pressure (*p* < 0.0001), 24-h SBP (*p* < 0.001), and 24-h DBP (*p* < 0.001). Furthermore, preterm-born adults showed higher fasting glucose (*p* = 0.01), fasting insulin (*p* = 0.002), Homeostasis Model Assessment-Estimated Insulin Resistance (HOMA-IR) index (*p* = 0.05), and total cholesterol levels (*p* = 0.05) compared to adults born at term. This meta-analysis established preterm birth as a significant, independent risk factor for metabolic syndrome and cardiovascular disease in adulthood [50,104,105].

#### 4.3.3. Very Low Birth Weight Adults

The New Zealand 1986 VLBW Study by Darlow et al. [87] assessed 229 VLBW survivors (birth weight < 1500 g) and 100 controls at ages 27–29 years. VLBW adults were significantly shorter than controls, with males showing significantly higher systolic blood pressure (*p* = 0.028). While the overall prevalence of metabolic syndrome did not differ significantly (22.2% vs. 11.1% for males; 12.8% vs. 13.1% for females), logistic regression identified male sex, gestational age < 28 weeks, and BMI > 90th percentile at age 7–8 years as significant predictors of metabolic syndrome at ages 27–29. Importantly, the youngest and most premature survivors (gestational age < 28 weeks) were at particularly high risk, with odds ratios of 2–4 for metabolic syndrome, highlighting the dose–response relationship between degree of prematurity and long-term metabolic risk.

#### 4.3.4. Distinguishing Preterm Birth from IUGR Effects

A recent large cohort study from France (Amadou et al. 2025) [53], examining 30,295 adults from the Constances cohort, provided important insights into the distinct long-term profiles of preterm birth versus non-preterm low birth weight (LBW). Non-preterm LBW is used here as a proxy for intrauterine growth restriction (IUGR); it is an imperfect one, since it will include constitutionally small infants and exclude growth-restricted infants born above 2500 g (Table 1). Preterm birth was associated with obesity (RR 1.25; 95% CI 1.08–1.46), hypertriglyceridemia (RR 1.23), high LDL-cholesterol (RR 1.16), high blood pressure (RR 1.22), metabolic syndrome (RR 1.35; 95% CI 1.06–1.71), and non-alcoholic fatty liver disease (RR 1.26). By contrast, non-preterm LBW (i.e., IUGR) was associated with prediabetes/diabetes (RR 1.30) and hypertension (RR 1.22) but—strikingly—with reduced risk of obesity (RR 0.83) and abdominal obesity (RR 0.84). This suggests that preterm birth and IUGR, while both conferring cardio-metabolic risk, have distinct long-term phenotypes: preterm birth was associated in this cohort with excess fat storage and the full metabolic syndrome cluster in adulthood, while IUGR was associated particularly with glucose dysregulation and hypertension without the same degree of excess adiposity. These are findings from a single volunteer cohort with self-reported birth data and have not yet been replicated; as discussed in Section 4.4.3 (“Differences Between Preterm and IUGR Catch-Up Growth”), they also sit uneasily with meta-analytic evidence that body size in preterm-born individuals is lower than that of term-born peers throughout childhood [106]. These differences have important implications for tailored surveillance and intervention strategies in adulthood.

### 4.4. Postnatal Growth and Nutrition

#### 4.4.1. Excessive Early Growth and Cardio-Metabolic Risk: Contemporary Epidemiological Evidence

An association between faster weight gain in infancy and childhood and later obesity has been reported in at least 21 studies across different populations and age groups [107,108]. These effects are observed for both linear growth and weight gain from as early as the first month of life, in both high-income and low-income countries, and even in predominantly breastfed populations.

Key observations include dose–response relationships between growth velocity and later disease risk; effects present across different ethnic and socioeconomic groups; and an asymmetric risk pattern where the combination of low birth weight followed by rapid postnatal growth confers the greatest cardio-metabolic risk—a pattern classically described in the Helsinki Birth Cohort, where the thinnest infants at birth who gained the most in BMI by age 11 had a fivefold elevated risk of coronary heart disease as adults [86]. These associations are observational and open to the confounding and over-adjustment described in Section 4.5. In particular, the excess risk attributed to low birth weight followed by rapid gain is estimated in models that condition on later body size, and faster gain is itself in part a mediator of attained size [90,91].

Some randomized evidence, derived mainly from term-born infants, is consistent with a causal effect of early nutrient intake on later outcomes: term infants born small for gestational age who were randomized to a nutrient-enriched formula had higher blood pressure six to eight years later [36], and infants randomized to nutrient-enriched formulas show metabolic effects that warrant follow-up despite short-term growth advantages [32]. These findings underlie the “early protein hypothesis”. Higher protein intake in infancy is associated with faster weight gain [84,85], and it has been proposed that it also alters metabolic programming and raises the later risk of obesity; the proposed mechanism is stimulation of insulin and insulin-like growth factor 1 (IGF-1) secretion, promoting early adipogenesis [109]. Nutrient-enriched formulas successfully assist preterm infants, and separately term infants born small-for-gestational-age (SGA), in reaching normal growth milestones. It has been hypothesized that rapid catch-up growth of this kind promotes preferential visceral fat deposition; the supporting human evidence is observational, and the mechanism has been demonstrated directly only in animal models [12,33,34,110]. Accelerated early growth has also been associated with a higher risk of developing type 2 diabetes later in life [110,111]. Healthcare providers must therefore weigh immediate growth needs against possible future metabolic risks in these populations, while recognizing that catch-up growth in preterm and growth-restricted infants is associated with better neurodevelopmental outcome and is in general desirable [13,112].

Human milk has a lower protein concentration than infant formula, and its protein content declines over the course of lactation, broadly matching the requirements of the healthy term infant. This does not hold for the very preterm infant, in whom unfortified human milk does not meet protein or mineral requirements and fortification is necessary (Section 5.3). Breastfed term infants typically grow more slowly in infancy [113]. Whether they have a lower BMI in the long term is less certain: in the largest randomized trial in this field, a breastfeeding-promotion intervention that substantially increased the duration and exclusivity of breastfeeding did not reduce adiposity at 6.5 years [114]. Lower protein intake is only one of several explanations for this difference, and possibly not the dominant one. Feeding from a bottle, whether of formula or of expressed human milk, is itself associated with greater weight gain than feeding at the breast, because the caregiver rather than the infant controls the volume taken and there is a well-documented tendency to encourage the infant to finish the bottle, overriding satiety cues [115,116]. In addition, powdered formula is frequently reconstituted more concentrated than the manufacturer specifies, so that the energy and protein actually delivered exceed the intended values [117,118]. Differences in growth between breastfed and formula-fed infants therefore reflect the mode of feeding and the accuracy of preparation as well as the composition of the milk. In recent years, term infant formula manufacturers have tended to move towards reducing protein content closer to human-milk levels to mitigate these risks [119].

#### 4.4.2. Postnatal Nutrition and Cardio-Metabolic Risk in VLBW Adults

A Finnish cohort study (Suikkanen et al. 2018) [88], following 166 VLBW adults (born 1978–1985) from the Helsinki Study of VLBW Adults, examined the relationship between early postnatal nutrition and cardio-metabolic risk factors in young adulthood. According to the “early protein hypothesis”, while high early protein intake is critical for preterm infants to optimize lean body composition, it must be carefully balanced to avoid metabolic overload. High protein and energy intake during the first weeks of life in very low birth weight infants predicts higher insulin resistance in young adulthood, rather than improving overall cardio-metabolic profiles [88]. Conversely, in full-term infants, lower protein exposure, such as that found naturally in breastmilk, has been associated with slower weight gain and lower later obesity risk [109]. Furthermore, poor-quality diets later in childhood exacerbate these risks by worsening blood pressure, triglycerides, and insulin resistance in young adults [85]. Although higher protein intake during the first weeks of life predicted a healthier body composition in adulthood, the findings of Suikkanen et al. [88], which are open to confounding by indication (Section 4.5), are consistent with the concept that the quality of early nutritional support, and protein intake in particular, may shape long-term cardio-metabolic outcomes, providing one of the rare direct links between documented neonatal nutrition and adult cardio-metabolic phenotype in humans.

#### 4.4.3. Catch-Up Growth: Benefits and Risks

##### The Catch-Up Growth Paradox

Catch-up growth, the accelerated growth following a period of restricted growth, represents one of the most complex and clinically challenging aspects of developmental programming. While rapid postnatal growth supports survival and neurodevelopment in growth-restricted infants, observational evidence suggests that this same rapid growth may increase long-term metabolic disease risk [36,120]. Two qualifications apply. First, catch-up growth in preterm and growth-restricted infants is associated with better neurodevelopmental outcome and is regarded in current expert guidance as a desirable recovery rather than a risk to be avoided [13,112]. Second, because faster weight gain is necessarily a mediator of attained size, the observational literature does not establish that accelerated rather than gradual gain, or gain in infancy rather than in later childhood or adolescence, carries independent risk [12,91]. The paradox addressed in this review is therefore a clinically important question whose answer is not yet established, rather than a demonstrated trade-off. The challenge lies in understanding the optimal balance between promoting adequate growth for immediate health and neurodevelopmental benefit while minimizing any long-term metabolic consequences [32,112,121,122].

##### Timing of Catch-Up Growt

The timing of catch-up growth appears to be critical in determining long-term health consequences. The “postnatal growth acceleration hypothesis” suggests that preterm infants and especially IUGR infants who demonstrate accelerated growth within the first two weeks of life have the greatest risk for endothelial dysfunction in adolescence and insulin resistance, compared to infants growing at a slower rate [11,32]. However, early catch-up growth that takes place within the first few months of life appears to be more beneficial than later catch-up growth, which may be more harmful [8,12,123,124]. This observation has important implications for nutritional management strategies in preterm infants and those with IUGR.

##### Pattern of Catch-Up Growth

The pattern of catch-up growth also appears to influence long-term outcomes. Catch-up growth occurring predominantly in height (linear growth) appears more favorable than catch-up growth occurring primarily in weight [125,126]. In a preterm cohort, Belfort et al. found that greater linear growth predicted better cognitive outcome without a corresponding increase in later adiposity, whereas weight gain in excess of linear growth predicted later overweight [127]. The practical implication is not that body composition should be measured routinely, but that length should be measured accurately with a length board at defined intervals and plotted alongside weight and head circumference, so that weight gain is interpreted in relation to linear growth rather than in isolation. This is an inexpensive and readily implemented change to standard practice (see also Section 4.4.3, “Quality of Growth and Body Composition”, and Section 7.4).

##### Quality of Growth and Body Composition

Recent research has emphasized the importance of body composition in assessing the quality of growth [8]. The distinction between lean tissue gain and adipose tissue accumulation is conceptually important for understanding the long-term health implications of different growth patterns [128,129]. At term-equivalent age, preterm infants have a higher fat mass percentage and a lower fat-free mass than infants born at term. The magnitude and the persistence of this difference should not be overstated. Pooling body composition measurements from 110 air-displacement-plethysmography studies (2855 preterm and 22,410 term infants) and 28 dual-energy X-ray absorptiometry studies, Hamatschek et al. found that the difference in percentage body fat present at term-equivalent age (16% in preterm versus 11% in term infants) had resolved by 52 weeks postmenstrual age (PMA), at which point the two groups reached 24% and 25% respectively [130]. The deficit in absolute fat-free mass is more persistent and has been reported to remain at four months corrected age, but the excess adiposity that has attracted most clinical attention is largely a transient feature of the first months of life [130,131]. Whether the body composition profile at term-equivalent age carries any independent long-term consequence, once subsequent growth is taken into account, has not been established.

Rapid and marked fat accumulation during catch-up growth may improve thermoregulation and increase energy stores, facilitating better adaptation to extrauterine life. However, this same fat accumulation, particularly central adiposity, may contribute to insulin resistance and cardiovascular disease risk in later life [8,12,124,132]. Air displacement plethysmography, dual-energy X-ray absorptiometry and skinfold-based estimation can quantify body composition in preterm infants and are valuable research tools. They have not, however, been shown to improve clinical outcomes when used for routine monitoring; they are costly and time-consuming, and normative data for the preterm population remain limited [133]. On current evidence they belong in research protocols and in selected clinical circumstances rather than in routine care, and accurate serial measurement of length remains the more practical and better-supported means of assessing the quality of growth [127,133].

##### Differences Between Preterm and IUGR Catch-Up Growth

While both preterm and IUGR infants commonly exhibit catch-up growth, the mechanisms and programming implications are hypothesized to differ; the account that follows is a proposed model rather than an established one. IUGR infants have experienced chronic intrauterine nutrient restriction, with an organ-sparing response that has been interpreted as calibrating the metabolic phenotype for a nutrient-poor environment. It has been proposed that when these infants encounter normal postnatal nutrition, the mismatch between the programmed physiology and the actual environment drives adipose tissue accumulation and metabolic dysregulation. Appropriate for gestational age (AGA) preterm infants, by contrast, have typically not experienced intrauterine nutritional restriction as such, but rather an abrupt disruption of the normal developmental sequence—losing the benefits of third-trimester placental nutrient delivery and facing metabolic demands for which they are physiologically unprepared.

Two recent lines of evidence caution against extending this model too far. In a systematic review and meta-analysis restricted to infants born preterm, Elmrayed et al. found that SGA status at preterm birth was not associated with greater adiposity in later childhood, a finding difficult to reconcile with a simple mismatch account in this population and one that questions whether the Barker hypothesis, derived from term-born growth-restricted infants, extends to preterm birth at all [134,135]. In an individual-participant-data meta-analysis of 253,810 singletons from 16 birth cohorts, Vinther et al. found that individuals born preterm were on average lighter and shorter than those born at term throughout childhood, with the difference narrowing during adolescence so that body size converged towards, rather than exceeded, that of the term-born population [106]. Preterm-born individuals therefore cannot be regarded as more prone to obesity in childhood. The adult excess risk of obesity reported in the Constances cohort by Amadou et al. [53] is better understood as emerging in adult life than as the continuation of a childhood trend, and the divergence between preterm and IUGR phenotypes described in that single cohort should be regarded as a finding requiring replication rather than as a settled distinction.

### 4.5. Interpreting the Evidence: Confounding, Over-Adjustment and Residual Bias

The evidence assembled in this section carries a structural limitation that constrains every conclusion drawn from it. Randomizing pregnant women or their infants to under- or over-nutrition is not ethically possible, so, with the exception of neonatal dietary trials such as those of Lucas [32,89,136], the human evidence base for developmental programming is observational. Three specific threats deserve explicit acknowledgement.

Confounding by social determinants of health. The social determinants of health—income, education, housing, food security, occupation and access to care—independently predict both faster infant weight gain and the adult outcomes reviewed here, namely higher blood pressure, cardiovascular disease and type 2 diabetes [137,138]. They are also strong determinants of preterm birth and of fetal growth restriction. A study that does not measure and adjust for them, or that adjusts using a single crude proxy such as maternal education, will attribute to early growth a portion of risk that in fact belongs to the social context in which that growth occurred. Maternal pre-pregnancy overweight and obesity [139] and maternal hypertensive disease of pregnancy [140] act similarly: each predicts both the exposure and the outcome, and each is inconsistently measured across the cohorts cited above.

Over-adjustment for attained body size. A more subtle and, in this literature, more pervasive problem is adjustment for weight or body mass index measured after the exposure. Later body size lies on the causal path between early growth and adult cardio-metabolic outcomes; conditioning on it removes precisely the effect the analysis is trying to estimate, and can additionally induce collider bias if unmeasured causes of both body size and the outcome exist [90,91]. Directed acyclic graphs make this explicit and have been recommended as a routine step in the design of pediatric observational studies [90]. Several of the studies summarized in Table 3 adjust for, or condition on, childhood or adult BMI; where they do, the reported association should be read as a conditional estimate rather than a total effect, and it is likely to be distorted, in this literature characteristically in the direction of increasing the apparent association between small size at birth and later outcome [90,91]. Table 3 flags the adjustment strategy of each study so that readers can weigh the estimates accordingly. A formal stratification of the whole literature into over-adjusted and non-over-adjusted strata, with separate pooled estimates, would be valuable, but it requires the protocol-driven search, risk-of-bias assessment and quantitative synthesis of a systematic review, and lies beyond what a narrative review can deliver; we identify it in Section 8.5 as a research priority.

Selective survival and confounding by indication. Adult cohorts of preterm survivors describe those who lived to be studied. In cohorts born in the 1970s and 1980s, survival at the lowest gestational ages was low and strongly patterned by birth weight, sex and social circumstances, so the surviving cohort is not a random sample of those born. Within the neonatal period, nutritional intake is not assigned at random but tracks illness severity, so associations between recorded neonatal intake and later outcome—as in Suikkanen et al. [88]—are open to confounding by indication.

Consequences for the conclusions of this review. Taken together, these considerations argue for reading the associations described above as hypotheses of varying strength rather than as established causal effects, and they explain why the clinical recommendations in Section 7 are anchored on society guidelines and randomized evidence wherever such evidence exists, rather than on the observational literature. They also bear directly on a specific question addressed in Section 4.4.3 (“Differences Between Preterm and IUGR Catch-Up Growth”): whether the Barker hypothesis, derived from term-born growth-restricted infants, extends to infants born preterm. Recent evidence suggests that it may not [134,135].

## 5. Special Nutritional Considerations for Preterm Infants

### 5.1. Unique Nutritional Challenges

Preterm infants face distinctive nutritional challenges that distinguish them from term infants, even those born at term with IUGR [7,8]. These include: disruption of normal organ developmental processes at a critical time; immature metabolic pathways with limited capacity to process and utilize certain nutrients; substantially increased requirements for energy, protein, and micronutrients to support both catch-up growth and ongoing development; feeding difficulties related to oro-motor immaturity and gastro esophageal reflux; and gastrointestinal immaturity including impaired motility, increased intestinal permeability, and heightened risk for necrotizing enterocolitis (NEC).

Poor postnatal growth in very preterm infants has commonly been described as extrauterine growth restriction (EUGR), most often defined as weight below the 10th percentile of an intrauterine reference at 36 weeks postmenstrual age or at discharge—a definition under which up to 90% of extremely preterm infants are classified as affected [141]. Both the term and this definition are now contested, and we use them here only to describe the earlier literature. The 2022 ESPGHAN position paper on enteral nutrition in preterm infants does not endorse EUGR as a diagnostic category [142]. The objection is a substantive one: a threshold applied at a single time point cannot distinguish the physiological downward centile crossing that follows the obligatory postnatal contraction of extracellular fluid, and that occurs in healthy preterm infants, from a genuine nutritional deficit. Consistent with this, weight below the 10th percentile at 36 weeks or at discharge has not been consistently associated with neurodevelopmental outcome once gestational age, illness severity and social circumstances are accounted for [143,144,145]. Change in weight and length z-score across the admission, interpreted together and against an appropriate reference, describes growth more informatively than any single-time-point cut-off, and that is the framing used in the remainder of this review. The adequacy of nutritional practice is better judged by whether delivered intakes meet recommended targets [142] than by the proportion of infants falling below a percentile line.

Supplementary Figure S2 summarizes the nutritional care of the preterm infant: the short-term goals that nutritional management must meet, the long-term cardio-metabolic associations that have been reported but not established as causal, and the four practical steps set out in Section 7.3, each annotated with the nature of its supporting evidence. It is a schematic aid to the text and does not imply that the long-term risks shown are likely or established.

### 5.2. Parenteral Nutrition: Early Provision and Composition

Adequate nutrient supplies shortly after preterm birth are essential for reducing the risk of suboptimal postnatal growth and adverse neurodevelopmental outcomes [146]. In the preterm infant, the brain is metabolically the most demanding organ for its function and programmed growth and maturation. Head circumference, linear growth, and weight gain are independently associated with neurodevelopmental outcome [147]. This implies early administration of protein—the primary driver of anabolic growth [14,15,16,148,149]—through parenteral nutrition until adequate enteral nutrition can be established [150,151,152,153,154].

Current evidence from ESPGHAN and ESPEN guidelines [155] recommends starting parenteral amino acid provision from birth, targeting intakes of 3.5 g/kg/day with energy provision of 90–120 kcal/kg/day. Early provision of amino acids, at doses of 1.5 g/kg/day from day 1 of life, has been shown to be safe and significantly improves nitrogen balance compared to dextrose-only solutions [15,16]. Higher amino acid intakes (>3 g/kg/day) in the first week of life are associated with improved early growth and neurodevelopmental outcomes [15,16].

The current consensus source for enteral nutrient intakes in this population is the 2022 position paper of the ESPGHAN Committee on Nutrition and invited experts [142], which addresses preterm infants with birth weight below 1800 g and provides graded conclusions and recommendations on protein, energy, lipid, carbohydrate, mineral and micronutrient intakes, on human milk and fortification, and on feed advancement [142]. That document is the primary guideline basis for the enteral recommendations set out in Section 7.3, and it should be noted that its authors explicitly identify several of its recommendations as resting on limited evidence and as requiring further trials with long-term functional outcomes.

A recent study by Lygerou et al. [156] examining 174 neonates in two NICUs demonstrated that early achievement of a positive energy and protein balance, based on ESPGHAN guidelines, is associated with better attained weight z-score at discharge. In view of the reservations set out in Section 5.1, we describe this outcome as attained weight z-score rather than as prevention of EUGR. In extremely preterm infants (<28 weeks), achieving a positive protein balance by day 14 was an independent predictor of improved weight z-scores at discharge.

### 5.3. Enteral Nutrition: Human Milk as First Choice

Human milk remains the feeding of choice for preterm infants [146,157,158]. The benefits are greatest for the mother’s own milk, and active support for mothers to express and provide milk for their infants is the principal means of achieving them [142,158]; in healthy term mother–infant pairs, randomized trials have shown that structured breastfeeding support increases the duration and exclusivity of breastfeeding [159]. In observational studies of preterm infants, the provision of mother’s own milk has been associated with maternal and social circumstances: it was less frequent among younger mothers, single mothers, mothers who smoked or had a substance use disorder, and families with lower household income or living in higher-poverty neighborhoods [160,161,162], circumstances that are themselves associated with infant outcomes. Part of the association between human-milk feeding and later outcomes may also reflect the social and health advantages that enable mothers to provide milk, and should be interpreted with that in mind (Section 6). Its benefits extend beyond macronutrient delivery to encompass a complex bioactive milieu that modulates gut microbiome colonization, immune programming, and neurodevelopment. Specific benefits documented in preterm infants include significantly reduced risk of necrotizing enterocolitis [14,157], reduced rates of late-onset sepsis, improved retinal vascularization, and better long-term neurodevelopmental outcomes [163].

However, unfortified human milk does not meet the elevated nutritional requirements of very preterm infants. Human milk fortification with multi-component fortifiers—providing additional protein, energy, calcium, phosphorus, and micronutrients—is standard care for preterm infants below 32–34 weeks. Despite the widespread use of standard fortification, significant variability exists in the macronutrient content of both maternal and donor human milk, contributing to nutritional uncertainty. Individualized (targeted or adjustable) vs. standard fortification—where milk analysis guides individualized fortification—is under investigation and shows promise for improving growth outcomes [164].

In the absence of maternal milk, pasteurized donor human milk is preferred over preterm formula as it retains many bioactive components of fresh milk while substantially reducing NEC risk. After discharge, the evidence that nutrient-enriched post-discharge formula improves growth or later outcomes is inconsistent, and current guidance favors individualized decisions based on serial growth assessment rather than routine use for a fixed period [142,165,166].

### 5.4. Key Micronutrients and Bioactive Components

Beyond macronutrients, several specific dietary components are of particular importance for preterm infant programming.

Long-chain polyunsaturated fatty acids (LC-PUFAs): DHA (docosahexaenoic acid) and ARA (arachidonic acid) are critical for brain development, retinal function, and immune modulation. Human milk is the principal source of these fatty acids for preterm infants. DHA levels in human milk decline rapidly postpartum, and maternal DHA supplementation can maintain higher milk DHA concentrations. In preterm infants, higher DHA intake is associated with improved visual acuity, white matter microstructure on MRI, and neurodevelopmental scores. Nava et al. [163] reviewed evidence that adequate DHA provision during NICU hospitalization is associated with better grey and white matter development and improved neurological outcomes in childhood (higher IQ and academic scores).

Human milk oligosaccharides (HMOs): HMOs are the third most abundant component of human milk and serve as prebiotics promoting Bifidobacterium colonization, providing immunomodulatory effects, serving as direct anti adhesive antimicrobials, and potentially acting as signaling molecules for intestinal development. The HMO 2’FL (2’-fucosyllactose) and other specific HMO structures have been associated with lower odds of NEC [163]. Formulas supplemented with HMOs show promise in approximating the microbiome-shaping benefits of human milk.

Vitamin D: Preterm infants are at high risk for vitamin D deficiency owing to immature stores, limited exposure to sunlight, and impaired 25-hydroxylation. Adequate vitamin D status is important not only for bone mineralization but also for immune programming, respiratory function, and possibly long-term cardio-metabolic programming [167].

Iron: Iron is critical for brain development, myelination, and energy metabolism [163]. Preterm infants have reduced iron stores and are at risk for iron deficiency anemia; however, excessive iron supplementation in the presence of infection can worsen oxidative stress. Current guidelines recommend supplemental iron starting at 2 weeks of age for enterally fed preterm infants.

### 5.5. Neurodevelopmental Outcomes and Nutritional Support

Neurodevelopmental impairment, including cognitive delay, motor abnormalities, behavioral problems, and increased rates of autism spectrum disorder and attention deficit hyperactivity disorder, is one of the most significant long-term burdens of prematurity. Nutrition plays a central role in supporting the vulnerable preterm brain during a period of extraordinary developmental activity [163].

In the preterm brain, white matter injury (periventricular leukomalacia) and dysmaturation are major concerns, reflecting both direct injury and altered qualitative development of white and grey matter. Nava et al. [163] summarized evidence that human milk, through its LCPUFA and HMO content, is associated with better grey and white matter development at brain MRI, which corresponds to better neurological outcomes in childhood. Higher nutritional intakes of calories and lipids during the NICU period appear associated with fewer severe brain lesions and better white and grey matter maturation.

The epigenetic clock findings of Gomaa et al. [68]—linking accelerated biological aging in preterm neonates to smaller brain volumes and worse neurodevelopmental outcomes at 18 months—suggest that epigenetic biomarkers may eventually guide individualized nutritional and neuroprotective interventions in the NICU. Nutritional strategies that attenuate epigenetic aging acceleration in preterm infants represent a promising but as yet unexplored therapeutic frontier.

Hortensius et al. [168] conducted a systematic review from bench to bedside of postnatal nutrition and brain development in preterm infants, confirming that higher protein and energy provision in the first weeks of life is associated with improved brain growth measures and neurodevelopmental outcomes, while emphasizing the importance of avoiding excessive weight gain that could counteract these benefits.

### 5.6. Long-Term Monitoring of Preterm Infants

Given the consistent observational evidence linking preterm birth to cardio-metabolic risk in adulthood, Morniroli et al. [7] advocate that prematurity should be considered a chronic disorder, with gestational age and birth history included in all medical records for patients of all ages when assessing chronic disease risk. This perspective calls for systematic long-term follow-up programs for preterm survivors, encompassing cardiovascular risk assessment, metabolic screening, and blood pressure monitoring well into adulthood.

Ruys et al. [148] emphasized the need to translate findings from nutritional intervention studies into daily clinical practice. They advocate for: (1) high protein-energy nutrition starting shortly after birth (energy > 100 kcal/kg/day, protein: energy ratio > 3 g/100 kcal); (2) discontinuation of the high-energy diet from 32–34 weeks PMA to prevent excess fat mass; and (3) individualized nutritional management guided by growth monitoring and body composition assessment. The second of these proposals has not been tested in trials and is not supported by current guidance [142]; it is identified as a research question in Section 8.4, and routine body composition assessment is not recommended (Section 7.4). Gounaris and Sokou [169] highlighted that changes in feeding protocols since 2000, especially for very premature neonates, have contributed significantly to improvements in survival and long-term morbidity.

## 6. Breastfeeding and Long-Term Health Outcomes

The associations summarized in this section come almost entirely from observational studies of term-born infants. Mothers who breastfeed, and who breastfeed for longer, differ systematically from those who do not in education, income and health behaviors [160,161,162], and these differences independently predict the outcomes considered here (Section 4.5). The one large randomized trial in this field, PROBIT, randomized maternity hospitals to a breastfeeding-promotion intervention; it achieved substantially longer and more exclusive breastfeeding but found no effect on adiposity or blood pressure at 6.5 years [114]. The estimates below should therefore be regarded as likely to include residual confounding. For the preterm infant, the benefits of human milk that rest on the firmest evidence are those described in Section 5.3.

### 6.1. Cardiovascular Benefits

Systematic reviews of observational studies have consistently reported that breastfeeding is associated with lower blood pressure in later life. Both systolic and diastolic blood pressure are reduced by 0.5–1.5 mmHg in breastfed compared to formula-fed infants [1,170,171]. While this effect size may appear modest at the individual level, it would, if causal, represent a meaningful population-level benefit; the PROBIT trial did not find an effect on blood pressure at 6.5 years [114]. Because all of the pooled studies were observational, these differences may reflect residual confounding by the maternal and social factors that predict both breastfeeding and later blood pressure (Section 4.5), and they should not be interpreted as an established effect of breastfeeding.

### 6.2. Metabolic Benefits

Breastfeeding has been associated with a lower risk of metabolic disorders [1]: a mean 3% reduction in insulin resistance; a mean improvement of 0.17 mmol/L (3.06 mg/dL) in glucose tolerance; and a 39% reduction in diabetes risk (OR 0.61) according to meta-analyses of seven observational studies [172]. These estimates have not been confirmed in a randomized trial, and the cohorts on which they rest were not able to exclude confounding by maternal socioeconomic position, maternal BMI and maternal diabetes, each of which is associated with both breastfeeding and offspring metabolic risk (Section 4.5). Proposed mechanisms for these associations include reduced growth velocity (compared to formula feeding), different hormonal responses to human milk feeding, the presence of bioactive components (including adipokines, hormones, and growth factors) in human milk, and favorable gut microbiome programming by HMOs.

### 6.3. Obesity Prevention

Systematic reviews of observational studies have reported that breastfeeding is associated with a 20% or greater reduction in obesity risk, with longer duration of breastfeeding associated with progressively lower risk [1,173,174]. This association has been reported to persist into adulthood, but it was not reproduced in the PROBIT trial [114] and is likely to be at least partly explained by confounding in observational studies. Part of the difference attributed to breastfeeding may also reflect the mode of feeding rather than the milk itself. Feeding from a bottle, whether of formula or of expressed human milk, makes it possible to over-feed: the caregiver rather than the infant controls the volume taken, there is a well-documented tendency to encourage the infant to finish the bottle, and bottle feeding has been associated with greater weight gain in infancy [115,116]. Proposed explanations for any true effect include the lower growth velocity of breastfed infants, who regulate their own intake (Section 4.4.1), and effects of human milk components on satiety, adipogenesis and microbiome composition.

### 6.4. Cholesterol and Lipid Profiles

Cholesterol concentrations are consistently lower in exclusively breastfed infants compared to formula-fed infants (mean difference −0.15 mmol/L [−5.80 mg/dL]) [1,175]. Paradoxically, breastfed infants have higher cholesterol levels during infancy itself, and it has been proposed that this early exposure “trains” cholesterol metabolism—a finding consistent with the hypothesis that early high cholesterol exposure programs more efficient regulation in adult life. This difference may contribute to long-term cardiovascular health benefits, though the mechanisms require further clarification. As in the preceding subsections, these findings come from observational studies and may be confounded by maternal and social factors (Section 4.5), and the proposed programming of cholesterol metabolism has not been demonstrated directly in humans.

### 6.5. Neurodevelopmental Benefits

Human milk feeding is associated with a 3–7 point IQ advantage in term infants and even greater benefits in preterm infants [136]. These estimates are derived almost entirely from observational studies, in which maternal intelligence, education and socioeconomic position are strong confounders of both breastfeeding and child IQ (Section 4.5); they should therefore be interpreted with caution. Proposed mediators of these associations include several bioactive components: long-chain polyunsaturated fatty acids (LCPUFA), particularly DHA; human milk oligosaccharides (HMOs); growth factors, including IGF-1 and EGF; and the influence of human milk on gut–brain axis signaling. Randomized evidence that early diet can have lasting effects comes from trials of formula composition rather than of breastfeeding: even a brief period of dietary intervention after preterm birth (26–34 weeks of gestation) has been reported to affect later brain development and cognition. For example, in Lucas’s randomized trial, adolescents born at ≤30 weeks who had been fed a nutrient-enriched diet had a verbal IQ about 6 points higher at age 16 years [31,32,89]. Neuroimaging studies have reported corresponding differences in brain structure. According to Nava et al. [163], human milk feeding in preterm infants is associated with better grey and white matter development on MRI scans, which has been associated with higher IQ and academic scores in childhood. In an observational analysis in which the amount of milk received was not randomized, Isaacs et al. [136] found that the percentage of expressed maternal breast milk (EBM) in an infant’s diet correlates significantly with verbal intelligence (VIQ) and white matter volume (WMV) in adolescence. In adolescent boys, this correlation also extended to total brain volume (TBV) and overall IQ scores.

## 7. Practical Clinical Implications

### 7.1. Preconception and Prenatal Care

Optimal nutritional programming begins before conception and continues throughout pregnancy. Key evidence-based recommendations for preconception and prenatal care include [1,92]:

Maternal nutritional optimization before pregnancy: Achieving healthy BMI (18.5–24.9 kg/m^2^) before conception reduces risks of both IUGR (underweight) and gestational diabetes, preeclampsia, and offspring metabolic programming (overweight/obesity). Periconceptional supplementation with folic acid (400–800 mcg/day) reduces neural tube defects and may have broader epigenetic benefits. Iron, vitamin D, iodine, and omega-3 fatty acid status should also be assessed and optimized preconceptionally.

Pregnancy nutrition: A balanced and varied diet providing adequate micronutrients is the foundation of pregnancy nutrition. Only a modest increase in energy intake (approximately 0.8 MJ/d or 191 kcal/day) is needed in the third trimester [1]. Excessive caloric intake—particularly from ultra-processed foods—increases risks of gestational weight gain above recommended levels and programs offspring adiposity [94]. Minimizing ultra-processed food consumption before and during pregnancy, in favor of whole foods rich in dietary fiber, micronutrients, and healthy fats, is recommended.

Weight management during pregnancy: Appropriate gestational weight gain based on pre-pregnancy BMI is recommended (11.3–15.9 kg for normal BMI, 6.8–11.3 kg for overweight, 5.0–9.1 kg for obese women) [176,177]. Overweight and obese women should be encouraged to minimize weight gain rather than attempt weight loss during pregnancy, as energy restriction may harm fetal development [178,179,180].

### 7.2. Postnatal Feeding Strategies for Term Infants

#### 7.2.1. Exclusive Breastfeeding

Exclusive breastfeeding is recommended for the first 6 months of life, with continued breastfeeding alongside complementary foods thereafter [181,182,183]. On-demand feeding should be encouraged, and unnecessary formula supplementation avoided. Feeding should also be responsive. Caregivers should be supported to recognize and respond to the infant’s hunger and satiety cues, and not to press an infant to finish a predetermined volume. The WHO identifies responsive feeding as a core component of appropriate infant and young child feeding and sets it alongside dietary composition rather than subordinate to it [184]. Responsive feeding bears directly on the growth patterns discussed in Section 4.4.1, because feeding from a bottle transfers control of intake from the infant to the caregiver [115,116]. It applies equally to expressed human milk and to formula, and it remains relevant after discharge for infants born preterm, in whom anxiety about growth can encourage caregivers to override satiety cues. Healthcare professionals play a crucial role in successful breastfeeding initiation through education, support, and early lactation consultation [1].

#### 7.2.2. Complementary Feeding

Introduction of complementary foods after 6 months should be guided by developmental readiness rather than arbitrary age cutoffs. Early introduction of solid food before 15 weeks is associated with higher weight and greater body fat percentage at 7 years [185], likely reflecting the establishment of excessive appetite and growth patterns during a critical developmental window. Complementary foods should be nutritionally dense, low in added sugar and salt, and gradually diverse.

#### 7.2.3. Preschool Nutrition

The preschool period (1–5 years) is a critical window during which long-term dietary habits and adiposity trajectories are established [1,186,187,188]. High protein intake in the preschool years has been associated with a more than doubled risk of later obesity, plausibly mediated through IGF-1 stimulation and accelerated adipogenic differentiation [109,188]; the association is observational and susceptible to the confounding discussed in Section 4.5. This period is included here because it is when the growth trajectory established during neonatal care is either consolidated or corrected, and because preterm and IUGR survivors enter it already carrying the risk profile described in Section 4.3.

### 7.3. Nutritional Management of Preterm Infants

The nutritional management of preterm infants should be guided by the dual imperative of supporting immediate survival, growth, and neurodevelopment while minimizing long-term metabolic programming risks. Evidence-based recommendations include [88,142,146,148,189]:

- **Early parenteral nutrition (PN)** *[Guideline]:* Initiate parenteral amino acids (at least 1.5 g/kg/day) from the first day of life, increasing to 2.5–3.5 g/kg/day from the second day and should be accompanied by non-protein intakes of more than 65 kcal/kg/day and adequate micronutrient intakes [155]. Provide intravenous lipid emulsions, with composite emulsions preferred over pure soybean oil, up to 4 g/kg/day [190]. Target parenteral energy provision of 90–120 kcal/kg/day in the stable growing phase [191].
- **Enteral Nutrition (EN)–Enteral Feeding** *[Guideline]:* Target full enteral nutrition by 2–3 weeks of age for most preterm infants, with enteral intakes as recommended by ESPGHAN (2022): protein 3.5–4.0 g/kg/day and energy 115–140 kcal/kg/day [142]. Protein intakes above this range are not supported by current evidence. However, in special cases protein intake may be further increased up to 4.5 g/kg/day where growth is slow, provided protein quality is good, concomitant energy and other micronutrient intakes are optimal, and there are no other causes for suboptimal growth. In such cases energy intakes of more than 140 kcal/kg/d may be needed, but should not exceed 160 kcal/kg/day. [142].
- **Human milk feeding** *[Guideline]* [142]: Use maternal milk exclusively when available, and provide active lactation support so that mothers can provide their own milk for their infants. When maternal milk is insufficient, supplement with pasteurized donor human milk rather than preterm formula, to preserve the microbiome-programming and NEC-protective benefits of human milk. Fortify human milk with multi-component fortifiers once tolerating full enteral feeds.
- **Individualized (targeted or adjustable) vs. standard fortification,** Section 5.3—*[Under investigation]:* Where milk analysis guides individualized fortification shows promise for improving growth outcomes [164].
- **Post-discharge nutrition** *[Guideline/Under investigation]*: The evidence that nutrient-enriched post-discharge formula improves outcomes is inconsistent, and current guidance recommends individualized decisions based on growth rather than routine use for a fixed period [142,165,166]. Re-evaluate growth pattern and nutritional needs at each follow-up visit, with accurate serial measurement of length and head circumference as well as weight (Section 7.4).
- **Responsive feeding**, post-discharge, Section 7.2.1—*[Guideline]* [184].

### 7.4. Growth Monitoring and Assessment

Growth monitoring is an essential component of nutritional management for both term and preterm infants. The pattern of growth is not only a marker of immediate well-being but has long-term implications for health risk [192]. WHO growth charts, based on slower-growing breastfed infants, are now the standard reference and help identify excessive growth velocity as well as growth faltering [7].

For preterm infants, Fenton [193] or INTERGROWTH-21st [194] charts should be used during NICU hospitalization to assess growth in the context of appropriate fetal growth references. Length should be measured accurately with a length board at defined intervals and plotted alongside weight and head circumference, since weight gain is interpretable only in relation to linear growth [127]. Body composition assessment using air displacement plethysmography (ADP), skinfold measurements or dual-energy X-ray absorptiometry (DEXA) yields information that weight alone does not [8], but it is costly and time-consuming, normative preterm data remain limited, and it has not been shown to improve outcomes when applied routinely. It is best regarded at present as a research tool and as an option in selected clinical circumstances, rather than as a component of standard care [133].

Individualized growth assessment should consider gestational age at birth, birth weight percentile, feeding method (breast vs. formula), family history, and environmental factors [7]. Upward centile crossing in weight, particularly when not accompanied by proportionate gains in length and head circumference, should prompt evaluation of overfeeding and the quality of growth.

### 7.5. Long-Term Cardio-Metabolic Follow-Up

Given the evidence for elevated cardio-metabolic risk in individuals born preterm or with IUGR, long-term follow-up programs are essential and currently underutilized. Such programs should incorporate regular blood pressure monitoring from early childhood; periodic assessment of fasting glucose, insulin, and lipid profile from adolescence; BMI and waist circumference tracking; and echocardiography for assessment of cardiac structure in those born at the earliest gestational ages.

Healthcare providers in adult medicine should be aware that patients born preterm or with low birth weight represent a population at elevated baseline cardiovascular risk, even in the absence of traditional adult risk factors. Gestational age at birth should be routinely recorded in adult medical histories, as it constitutes clinically relevant risk information analogous to family history of coronary heart disease [7].

## 8. Future Directions and Research Needs

### 8.1. Mechanistic Understanding

Key areas requiring further mechanistic investigation include: the reversibility of epigenetic modifications established in early life and the potential for nutritional or pharmacological interventions to correct adverse epigenetic programming; the specific critical windows of vulnerability for different organ systems; organ-specific programming effects and their relative contributions to overall cardio-metabolic risk; and the mechanisms of transgenerational transmission of programming effects, including germline epigenetic inheritance.

### 8.2. Epigenetic Biomarkers and Precision Nutrition

Epigenetic clocks and other DNA methylation-based biomarkers hold promise as tools for identifying infants at elevated risk for adverse long-term outcomes and for monitoring the impact of nutritional interventions on biological aging and development [68]. Future research should establish reference values for epigenetic age in preterm and IUGR infants; test whether specific nutritional interventions (e.g., higher LCPUFA provision, probiotic supplementation, targeted human milk fortification) attenuate epigenetic age acceleration; and validate epigenetic biomarkers as surrogate endpoints for long-term outcome studies. More broadly, these biomarkers place early nutrition within the emerging agenda of life-course longevity medicine, which seeks to extend gerodiagnostic and geroprotective strategies into the perinatal period (see Section 8.7) [195].

### 8.3. Gut Microbiome Interventions

The gut microbiome represents a modifiable mediator of developmental programming with significant therapeutic potential. Future research priorities include: optimization of prebiotic (HMO) supplementation to promote beneficial microbiome colonization in preterm infants; evaluation of specific probiotic strains (particularly Bifidobacterium species) for their ability to modulate metabolic programming; understanding the long-term microbiome and metabolic consequences of antibiotic exposure in early life; and elucidating the mechanisms linking specific microbial metabolites (e.g., short-chain fatty acids, secondary bile acids) to long-term cardio-metabolic programming.

### 8.4. Intervention Strategies and Clinical Trials

Development of evidence-based interventions to optimize early nutrition while minimizing long-term risks is a critical research priority. Needed are randomized controlled trials with long-term follow-up examining the metabolic consequences of different protein: energy ratios in preterm infants; evaluation of whether, and when, the balance of protein and energy should change after 32 weeks PMA, including the untested proposal that energy intake be reduced while protein intake is maintained (Section 5.6); testing of targeted human milk fortification versus standard fortification on body composition and long-term cardio-metabolic outcomes; and trials of preconception nutritional interventions in high-risk populations.

### 8.5. Long-Term Outcome Studies and Registry-Based Research

Longitudinal studies with extended follow-up periods into adulthood are essential for documenting cardio-metabolic outcomes in preterm and IUGR survivors; identifying critical periods for intervention; developing predictive models incorporating gestational age, growth patterns, and nutritional history; and evaluating the effectiveness of NICU nutritional interventions on adult health. Registry-based studies—exemplified by the Swedish, Finnish, and New Zealand cohorts—have proven invaluable and should be expanded to include detailed nutritional data from the perinatal period linked to long-term health outcomes.

A specific methodological priority is a systematic review of the association between early growth and adult cardio-metabolic outcomes that stratifies the literature by whether analyses adjusted for body size attained after the exposure, and that pools the two strata separately. Because attained body size lies on the causal path, the two strata answer different questions, and combining them—as most current syntheses do—yields estimates that are difficult to interpret [90,91]. Pre-specified directed acyclic graphs should accompany the analysis plan of future observational studies in this field [90].

### 8.6. Personalized and Sex-Specific Approaches

Emerging evidence suggests important sex differences in the long-term programming effects of preterm birth and early nutrition. Amadou et al. [53] found that preterm-born women have higher relative risks of hypertriglyceridemia and metabolic syndrome than preterm-born men. Darlow et al. [87] found sex differences in body composition and blood pressure outcomes in VLBW adults. Future research should explicitly account for sex as a biological variable in programming studies, and clinical guidelines should consider sex-stratified recommendations where evidence supports differential effects. Additionally, genetic background, including polymorphisms in key metabolic pathways, may modulate programming susceptibility and response to nutritional interventions, pointing toward the future of personalized early-life nutrition.

### 8.7. From Developmental Programming to Longevity Medicine: A Life-Course Convergence

The evidence synthesized in this review—that early nutrition and growth durably shape cardio-metabolic risk through epigenetic and structural programming—intersects directly with the rapidly expanding field of healthy-longevity, or geroscience, medicine [196,197]. Geroscience holds that biological ageing is modifiable and is a shared upstream driver of the chronic non-communicable diseases to which preterm and IUGR survivors are predisposed, and it implies that the trajectory of that ageing is set far earlier than the mid-adult life at which longevity medicine has traditionally intervened [196]. The maturation of epigenetic-clock technology has made this convergence measurable: DNA-methylation–based estimators now quantify biological and gestational age from the neonatal period [198,199], and, as discussed above (Section 3.2.4), extremely preterm infants already display accelerated epigenetic ageing that predicts adverse neurodevelopmental outcomes [68]. Building on this premise, an international consortium (PROSPER) has recently proposed relocating the starting point of longevity medicine to the preconception, intrauterine, and early-postnatal windows—precisely the periods addressed in this review—and embedding gerodiagnostic and geroprotective strategies within routine obstetric and pediatric care [195]. Within this framework, the nutritional principles advocated in this review—human-milk feeding, optimized protein–energy balance, and avoidance of excessively rapid catch-up growth—may be reconceived as among the earliest feasible interventions to preserve health span and functional “peak span.” Important caveats remain: normative age-metric reference curves and biomarkers validated specifically for the developing child are still lacking, current tools more convincingly extend health span than lifespan, and the use of early-life biological-age data raises ethical questions of privacy, labelling, and equitable attribution of responsibility. Nevertheless, positioning preterm and IUGR infants at the intersection of developmental programming and longevity science reframes neonatal nutrition not merely as short-term support but as a foundational determinant of lifelong biological ageing.

## 9. Conclusions

The relationship between early nutrition and long-term health represents one of the most important and clinically relevant paradigms in modern medicine. The evidence reviewed here is consistent with the hypothesis that nutrition during critical periods of development, from preconception through the first years of life, can durably alter physiological function and disease susceptibility, although the human evidence is largely observational and its strength differs considerably between exposures and outcomes.

For preterm infants and those with IUGR, this presents both challenges and opportunities. Adequate nutrition is essential for immediate survival and neurodevelopment, yet the long-term metabolic consequences of nutritional strategies must be carefully balanced. Preterm birth itself has been associated with ischemic heart disease, metabolic syndrome, hypertension and dyslipidemia in adulthood, in the strongest analyses independently of shared familial factors, conferring cardio-metabolic risks that are distinct from those associated with IUGR. These associations rest almost entirely on observational data and are open to confounding by social determinants of health and by maternal factors, and in many published analyses to over-adjustment for attained body size (Section 4.5). They are presented here as the best available evidence, not as established causal relationships, and the clinical recommendations that follow are anchored wherever possible on society guidelines and on randomized evidence.

The hypothesized mechanisms underlying developmental programming are multiple and probably interacting, encompassing epigenetic modifications (particularly DNA methylation and histone remodeling), structural organ deficits resulting from interrupted development, hormonal axis reprogramming, gut microbiome dysbiosis, and oxidative stress. The emergence of epigenetic clock technology provides new tools for quantifying biological programming in preterm infants and may eventually guide individualized interventions. These advances increasingly align developmental programming with the nascent field of longevity medicine, reframing neonatal nutrition as a determinant not only of childhood outcomes but of lifelong biological ageing (Section 8.7).

Key principles for clinical practice, graded in Section 7.3 by the strength of their supporting evidence, include: (1) attention to the preconception period as the earliest opportunity for nutritional programming intervention; (2) optimization of maternal nutrition and BMI before and during pregnancy; (3) early nutritional support for preterm infants in accordance with current parenteral and enteral nutrition guidelines, with adequate protein provision from birth; (4) support for mothers to provide their own milk for their infants, with human milk as the basis of preterm infant feeding; (5) support for responsive feeding, so that caregivers respond to the infant’s hunger and satiety cues rather than to a target volume, both during the birth admission and after discharge; (6) growth monitoring in which length and head circumference are measured accurately and plotted alongside weight, so that weight gain is interpreted in relation to linear growth rather than in isolation; (7) awareness that preterm birth is associated with increased cardio-metabolic risk in adulthood, warranting long-term surveillance; and (8) a multidisciplinary approach involving neonatologists, dietitians, pediatricians, and adult care providers to ensure continuity of nutritionally informed care.

The field of developmental and nutritional programming continues to evolve rapidly, with new insights emerging from advances in epigenomics, microbiome science, systems biology, and long-term outcome studies. As our understanding deepens, we will be better positioned to develop evidence-based strategies that optimize both immediate and long-term health outcomes for these vulnerable populations—and potentially to break the transgenerational cycles of disease that early nutritional adversity may set in motion.

## Figures and Tables

**Table 1:** Terms used in this review to describe the study populations, and the distinctions between them.

Term	Definition	What It Does and Does Not Tell You
Preterm birth	Birth before 37 + 0 completed weeks of gestation. Extremely preterm < 28 + 0; very preterm 28 + 0–31 + 6; moderate 32 + 0–33 + 6; late preterm 34 + 0–36 + 6 weeks.	A statement about gestational duration only. It says nothing about whether fetal growth was adequate.
Low birth weight (LBW) Very low (VLBW) Extremely low (ELBW)	Birth weight < 2500 g, Birth weight < 1500 g and Birth weight < 1000 g respectively, Irrespective of gestational age.	A weight category that conflates short gestation with restricted growth. In the historical cohorts on which the Barker hypothesis rests, gestational age was frequently unrecorded and LBW served as a proxy for growth restriction; in contemporary neonatal cohorts, most VLBW and ELBW infants are LBW because they are preterm, not because they are growth-restricted.
Small for gestational age (SGA)	Birth weight (and sometimes length or head circumference) below the 10th centile for gestational age and sex on a specified reference or standard.	A statistical description at a single time point, dependent on the chosen reference. It includes constitutionally small but healthy infants, and it misses growth-restricted infants whose birth weight remains above the 10th centile.
Fetal growth restriction (FGR)/intrauterine growth restriction (IUGR)	Failure of the fetus to achieve its genetically determined growth potential, most often from placental insufficiency. A pathophysiological diagnosis requiring serial fetal biometry and, in current definitions, Doppler criteria.	A statement about a process, not about a threshold. An IUGR fetus may or may not be born SGA; an SGA infant may or may not be growth-restricted. The two terms are used interchangeably in much of the older literature and this is a recognized source of confusion.
Asymmetric growth restriction	Disproportionate reduction in abdominal circumference relative to head circumference, reflecting redistribution of fetal blood flow (Section 4.2.2).	Describes the pattern, and by inference the timing and the placental mechanism, of restriction.
Extrauterine growth restriction (EUGR)	Historically, weight below the 10th centile of an intrauterine reference at 36 weeks postmenstrual age or at discharge.	A contested term not endorsed by ESPGHAN, and used in this review only when describing the earlier literature (Section 5.1).

These distinctions are maintained throughout the review. Where the primary source reported a population that does not map cleanly onto them—most commonly a cohort defined by low birth weight without gestational age, which necessarily mixes preterm and growth-restricted infants—this is stated at the point where the study is described, and the reader is asked to interpret the finding accordingly. The Barker cohorts and the Constances analysis of “non-preterm low birth weight” are the two principal instances.

**Table 2:** Proposed mechanisms of developmental programming: from early-life trigger to hypothesized long-term consequence.

Key Process/ Target	Key References	Early-Life Trigger	Molecular or Structural Change	Long-Term Consequence (Representative Evidence)
		**Structural & functional organ deficits (“reduced functional units”)**		
Kidney—nephrogenesis	[47,48,49,50]	Preterm birth (nephrogenesis completes 34–36 wk; >60% of nephrons form in the 3rd trimester)	↓ nephron endowment (up to one-quarter to one-third fewer) → compensatory hyperfiltration	Hypertension and chronic kidney disease; higher systolic/diastolic blood pressure in preterm adults
Pancreas—β-cell development	[51,52]	Under-nutrition/preterm birth during 3rd-trimester–early-postnatal window	↓ islet/β-cell number and insulin secretory capacity	Glucose intolerance and type 2 diabetes
Heart—cardiomyogenesis	[53]	Preterm birth; altered loading	↓ cardiomyocyte number; altered cardiac geometry	↑ risk of heart failure and arrhythmia in later life
Skeletal muscle—myogenesis	[53]	Fetal nutrient restriction	↓ muscle mass → ↓ basal metabolic rate & exercise capacity	Predisposition to obesity and insulin resistance
Liver—hepatic development	[53]	Fetal programming	Altered lipid-metabolism capacity	Non-alcoholic fatty liver disease (preterm RR 1.26)
Adipose tissue	[54]	Maternal obesity; late-fetal specification of adipocyte progenitors	Altered adipocyte progenitor pool and adipose-expansion trajectory	Accelerated early adiposity → childhood and adult obesity
		**Epigenetic mechanisms (mitotically heritable; no change in DNA sequence)**		
DNA methylation	[58,59,60,61,62,63,64]	Maternal diet/methyl-donor supply; famine	Altered CpG methylation of metabolic-gene promoters (e.g., IGF-2; agouti model)	Persistent altered gene expression and metabolic-disease susceptibility (Dutch famine: change measurable ~60 y later)
Histone modification	[65,66]	Under-nutrition; cofactor (acetyl-CoA, S-adenosyl-methionine) availability	Shifted H3K4me3 (active)/H3K27me3 (repressive) marks at metabolic promoters	Altered glucose-metabolism, adipogenic and inflammatory gene programs
Non-coding RNA	[67]	Maternal nutritional status; growth restriction	Differential miRNA/lncRNA expression in placenta and fetal tissues	Dysregulated insulin signalling, adipogenesis and inflammation
Epigenetic clocks	[68]	Extreme prematurity	Accelerated biological (PedBE) age at term-equivalent age	Smaller brain volumes and worse neurodevelopment—a risk biomarker
		**Hormonal, microbiome & oxidative mechanisms**		
Hormonal reprogramming	[71,72,73,84,85]	Early over- or under-nutrition; high early protein	Altered leptin/insulin set-points; programming of hypothalamic NPY–AgRP appetite circuits; ↑ IGF-1	↑ appetite and ↓ energy expenditure → obesity; the “early-protein” effect on weight gain and blood pressure
Gut-microbiome dysbiosis	[74,75,76,77]	Caesarean delivery, broad-spectrum antibiotics, delayed enteral feeds, prolonged parenteral nutrition	↓ diversity and Bifidobacterium; Enterobacteriaceae dominance; ↓ short-chain fatty acids	Intestinal barrier failure, systemic inflammation, adipose programming and insulin resistance; human-milk oligosaccharides are protective
Oxidative stress	[78,79,80,81,82,83]	Antioxidant immaturity, supplemental O_2_, infection, ischemia–reperfusion; maternal obesity/pre-eclampsia	ROS–antioxidant imbalance; perturbed nitric-oxide–ADMA axis	Maladaptive cardiovascular–kidney–metabolic programming → hypertension and chronic kidney disease

↑, increased; ↓, decreased; →, leading to; ADMA, asymmetric dimethyl-arginine; IGF-2, insulin-like growth factor 2; lncRNA, long non-coding RNA; miRNA, microRNA; NAFLD, non-alcoholic fatty liver disease; NPY–AgRP, neuropeptide-Y/agouti-related peptide; PedBE, pediatric buccal epigenetic clock; ROS, reactive oxygen species; SCFA, short-chain fatty acids. All entries are proposed mechanisms rather than demonstrated causal pathways. The supporting evidence is observational in humans and experimental only in animal models; it is summarized in the corresponding subsections of Section 3, where the primary sources are cited. Levels of supporting evidence differ substantially between rows and are discussed in the opening paragraph of Section 3.

**Table 3:** Selected human studies linking early-life exposures to long-term cardio-metabolic outcomes, with study design and adjustment for body size attained after the exposure.

Study (First Author, Year)	Population/Cohort (n)	Design & Exposure	Design Tier and Adjustment for Later Body Size	Principal Long-Term Findings
Barker, 1989–90 [21,23]	England & Wales districts (ecological)	Geographical correlation study; infant mortality as marker of early-life deprivation	Ecological (area-level) correlation—the weakest observational design; no individual-level adjustment possible, and area-level infant mortality is itself a proxy for deprivation. Hypothesis-generating only.	High infant-mortality districts (poverty, low birth weight) showed high coronary heart disease mortality 50–70 y later. Small size at birth linked to later hypertension, coronary heart disease and non-insulin-dependent diabetes—the foundation of the fetal-origins hypothesis.
Dutch Hunger Winter cohort [61,62,63,64]	Adults exposed in utero to the 1944–45 famine (rations 400–800 kcal/day)	Natural experiment; precisely timed prenatal undernutrition	Natural experiment; exposure timing is essentially unconfounded by family choice, which is the principal strength of this cohort. Selective survival of the famine and of the follow-up period is a limitation. Sibling comparisons were used for the methylation analyses.	Early-gestation exposure → higher adult BMI, cholesterol and coronary heart disease risk; late-gestation exposure → impaired glucose tolerance. ≈5% lower IGF-2 methylation was still measurable ~60 y later, specific to early-gestation exposure—evidence of durable epigenetic programming and critical windows.
Helsinki Birth Cohort [20,86]	Finnish men and women followed from birth	Longitudinal birth-cohort	Longitudinal cohort. The reported effect is a conditional-growth estimate: the risk attributed to “gaining the most BMI by age 11” is estimated conditional on birth size, so the estimate depends on the modelling choice and is not a simple contrast of exposed and unexposed groups.	The thinnest infants at birth who gained the most BMI by age 11 had a ~5-fold elevated risk of coronary heart disease as adults—low birth size plus rapid childhood weight gain confers the greatest risk.
Crump et al., 2019 [9]	Sweden—2,141,709 singletons born 1973–1994	National cohort; gestational age at birth; co-sibling analysis	National registry cohort with co-sibling analysis—the strongest design in this table for controlling shared familial and socioeconomic confounding. Adjusted for birth characteristics and sociodemographic factors, not for adult BMI, so not over-adjusted for the outcome pathway.	At ages 30–43 y, preterm birth (<37 wk) was associated with a 53% higher risk of ischemic heart disease (adjusted HR 1.53; 95% CI 1.20–1.94); early-term (37–38 wk) HR 1.19. Relative risk higher in women (1.93) than men (1.37). Not explained by shared familial factors—supporting a causal role for preterm birth.
Markopoulou et al., 2019 [50]	Meta-analysis—43 studies; 18,295 preterm vs. 294,063 term-born adults	Systematic review & meta-analysis	Meta-analysis of observational studies. Constituent studies differ in whether they adjusted for adult BMI; where they did, estimates for blood pressure and insulin resistance are conditioned on a variable that lies on the causal pathway and are likely distorted.	Preterm-born adults had significantly higher fat mass, systolic and diastolic (incl. 24-h) blood pressure, fasting glucose and insulin, HOMA-IR and total cholesterol than term-born adults—establishing preterm birth as an independent risk factor for the metabolic syndrome.
Darlow et al. (New Zealand 1986 VLBW study) [87]	229 VLBW survivors (<1500 g) + 100 controls, assessed at 27–29 y	Prospective cohort	Prospective cohort with contemporaneous controls. BMI > 90th centile at 7–8 y is entered as a predictor of adult metabolic syndrome; because childhood BMI lies on the causal pathway between the exposure and the outcome, the estimates for gestational age and sex are conditioned on it and are likely distorted. The analysis is best read as descriptive of a trajectory rather than as an estimate of an independent effect of prematurity.	VLBW adults were shorter; males had higher systolic blood pressure. Independent predictors of metabolic syndrome were male sex, gestational age < 28 wk and BMI > 90th centile at 7–8 y. The most premature (<28 wk) had OR 2–4 for metabolic syndrome—a dose–response with degree of prematurity.
Amadou et al., 2025 (Constances) [53]	France—30,295 adults	Cohort; preterm birth vs. non-preterm low birth weight (IUGR)	Large volunteer cohort; birth data are self-reported, and participation in Constances is socially patterned. Adjusted for age, sex and socioeconomic position; not adjusted for adult BMI in the obesity models.	Preterm birth: obesity RR 1.25, hypertriglyceridemia 1.23, high LDL 1.16, high blood pressure 1.22, metabolic syndrome 1.35, NAFLD 1.26. Non-preterm LBW/IUGR: prediabetes-diabetes RR 1.30 and hypertension 1.22, but reduced obesity (0.83) and abdominal obesity (0.84)—distinct long-term phenotypes.
Suikkanen et al., 2018 (Helsinki VLBW) [88]	166 VLBW adults born 1978–1985, with documented neonatal nutrition	Cohort linking recorded neonatal intake to adult outcomes	Cohort with prospectively recorded neonatal intake—unusual and valuable—but nutritional intake in the 1978–85 era was not randomized and tracked closely with illness severity, so confounding by indication is likely: the sickest infants received different nutrition and had different outcomes for other reasons.	High protein and energy intake in the first weeks of life predicted higher insulin resistance in young adulthood—one of the rare direct links between documented neonatal nutrition and adult cardio-metabolic phenotype, supporting the “early-protein” hypothesis.
Gomaa et al. [68]	35 very preterm neonates (24–32 wk)	Prospective; pediatric buccal epigenetic (PedBE) clock + brain MRI	Small prospective cohort (n = 35) with a surrogate biomarker endpoint. Hypothesis-generating; not powered for clinical outcomes.	Extremely preterm neonates (<28 wk) showed accelerated epigenetic ageing. Accelerated PedBE age at term-equivalent age independently predicted smaller cerebral volumes, slower brain growth and worse cognitive/language scores at 18 months—a candidate biomarker of neurodevelopmental risk.
Lucas — enriched vs. standard preterm formula [31,32,89]	Preterm infants (26–34 wk)	Randomised controlled trial with long-term follow-up	Randomized controlled trial with long-term follow-up—the only experimental evidence in this table and the highest-tier evidence in the review. Attrition over 16 years is the main threat to validity.	A brief early dietary intervention affected brain development and IQ, with a ~6-point IQ advantage at age 16 in the enriched-diet group—the experimental basis of nutritional “programming” in preterm infants.

Values are as reported in the cited studies. Bracketed numbers correspond to the reference list of the main manuscript. →, leading to; BMI, body mass index; CI, confidence interval; HR, hazard ratio; HOMA-IR, homeostasis-model-assessment insulin resistance; IUGR, intra-uterine growth restriction; LBW, low birth weight; LDL, low-density lipoprotein; MetS, metabolic syndrome; NAFLD, non-alcoholic fatty liver disease; OR, odds ratio; RR, relative risk; VLBW, very low birth weight. Adjustment for, or conditioning on, body size attained after the exposure (over-adjustment) tends to distort, and in this literature to increase, estimated associations between early-life exposures and later outcomes [90,91]; estimates from studies flagged in this respect in the fourth column should be interpreted accordingly.

## Data Availability

No new data were created or analyzed in this study. Data sharing is not applicable to this article.

## Supplementary Materials

The following supporting information can be downloaded at: https://www.mdpi.com/article/10.3390/nu18213542/s1, Figure S1: Schematic representation of the developmental origins of health and disease (DOHaD) hypothesis across the life course; Figure S2: Schematic summary of the considerations in the nutritional care of the preterm infant.

## Abbreviations

The following abbreviations are used in this manuscript:

ADMA	asymmetric dimethyl-arginine
ADP	air displacement plethysmography
ARA	arachidonic acid
BMI	body mass index
CI	confidence interval
CA	corrected age
DBP	diastolic blood pressure
DEXA	dual-energy X-ray absorptiometry
DHA	docosahexaenoic acid
DNA	deoxyribonucleic acid
DOHaD	developmental origins of health and disease
EBM	expressed breast milk
EN	enteral nutrition
ESPEN	the European society for clinical nutrition and metabolism
ESPGHAN	European society for pediatric gastroenterology, hepatology and nutrition
EUGR	extra-uterine growth restriction
GLP-1	glucagon-like peptide-1
GPCR	G-protein coupled receptor
HOMA-IR	homeostasis-model-assessment insulin resistance
HMOs	human milk oligosaccharides
HR	hazard ratio
HTN	hypertension
IGF-2	insulin-like growth factor 2
IQ	intelligence quotient
IUGR	intrauterine growth restriction
LBW	low birth weight
LC-PUFA	Long-chain polyunsaturated fatty acid
LDL	low-density lipoprotein
lncRNA	long non-coding RNA
MetS	metabolic syndrome
miRNA	microRNA
MRI	magnetic resonance imaging
NAFLD	non-alcoholic fatty liver disease
NEC	necrotizing enterocolitis
NICU	neonatal intensive care unit
NO	nitric oxide
NPY–AgRP	neuropeptide-Y/agouti-related peptide
OR	odds ratio
PedBE	pediatric buccal epigenetic
PMA	post-menstrual age
PN	parenteral nutrition
RNA	ribonucleic acid
ROS	reactive oxygen species
RR	relative risk
SAMe	S-adenosylmethionine
SBP	systolic blood pressure
SCFA	short-chain fatty acids
SGA	small for gestational age
TBV	total brain volume
VIQ	verbal intelligence quotient
VLBW	very low birth weight
WMV	white matter volume
wk	weeks
