# Supplementary material for: The Early Nutrition Paradox in Preterm and Intrauterine Growth Restricted (IUGR) Infants: A Critical Review of the Developmental Programming Hypothesis and the Limits of Its Evidence
Source: Nutrients. 2026 Oct 27;18(21):3542. doi: 10.3390/nu18213542 (PMC13765296; doi:10.3390/nu18213542)
Supplement: Supplementary file 1 [file nutrients-18-03542-s001.zip › nutrients-4498166-supplementary.pdf]

Supplementary Materials

The Early Nutrition Paradox in Preterm and Intrauterine Growth Restricted (IUGR) Infants: A Critical Review of the Developmental Programming Hypothesis and the Limits of Its Evidence

Arieh Riskin, Adir Iofe, Rasha Zoabi Safadi, Naama Tal and Ayala Gover

Nutrients, manuscript nutrients-4498166

Contents: Figure S1 (p. 2); Figure S2 (p. 3).

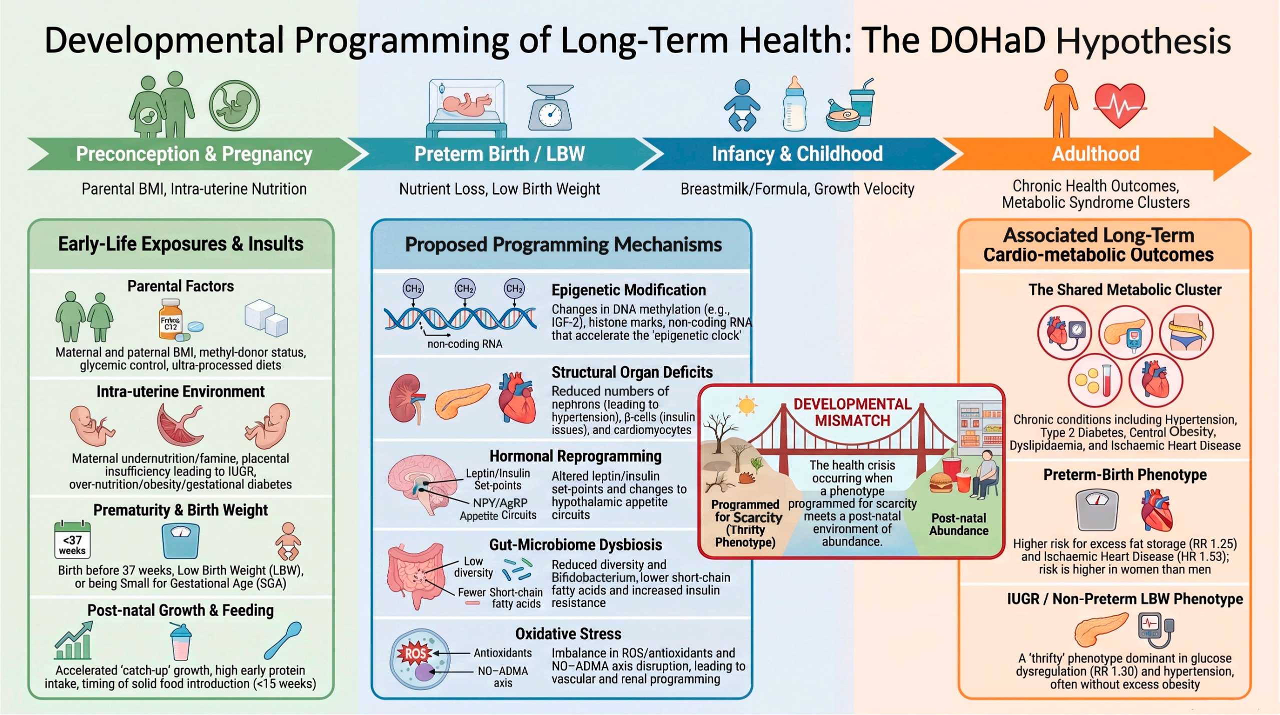

Hypothesized pathways: the supporting human evidence is observational. The adult phenotypes shown are those reported in a single cohort.

Figure S1. Schematic representation of the developmental origins of health and disease (DOHaD) hypothesis across the life course. The figure illustrates what the hypothesis proposes; it does not depict demonstrated causal pathways and should not be read as evidence for them. The human evidence bearing on each element is observational, of variable and often low strength, and vulnerable to confounding and to over-adjustment for attained body size (main text, Sections 1 [Scope, Literature Search and Appraisal of the Evidence], 3 and 4.5); several of the mechanisms shown rest mainly on animal studies. The figure does not show the factors that may confound these associations, notably the social determinants of health, maternal pre-pregnancy body mass index and maternal hypertensive disease of pregnancy, which are associated with preterm birth, fetal growth, early postnatal growth and adult cardio-metabolic outcomes alike. Parental factors are shown as early-life exposures, but they are themselves shaped by social and economic circumstances; the figure is not intended to attribute responsibility for these outcomes to parents. According to the hypothesis, exposures in successive periods (preconception and pregnancy, preterm birth or intrauterine growth restriction, and postnatal growth and feeding) act through the mechanisms shown in the central panel to influence adult cardio-metabolic risk. The "developmental mismatch" panel represents a hypothesis, not an established finding. The adult phenotypes shown on the right are associations reported in a single cohort (Amadou et al., 2025; reference [53] in the main text) that require replication; they are not established consequences of preterm birth or intrauterine growth restriction. RR, relative risk; HR, hazard ratio; HTN, hypertension; LBW, low birth weight; SGA, small for gestational age; IUGR, intrauterine growth restriction; NAFLD, non-alcoholic fatty liver disease; SCFA, short-chain fatty acids; ADMA, asymmetric dimethyl-arginine; ROS, reactive oxygen species.

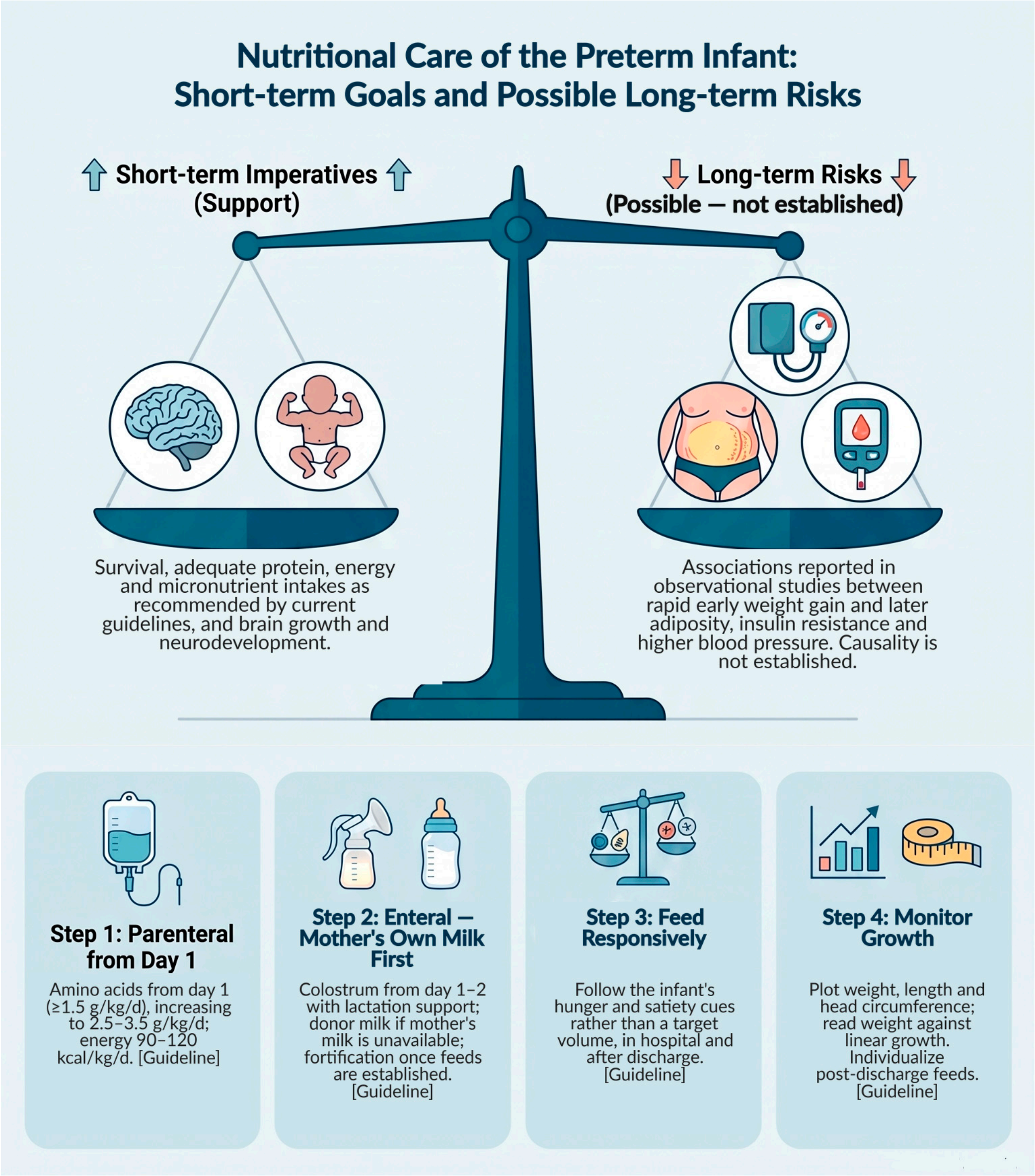

Figure S2. Schematic summary of the considerations in the nutritional care of the preterm infant. The left side shows the short-term goals of nutritional management: survival; intakes of protein, energy and micronutrients as recommended by current guidelines; and brain growth and neurodevelopment. The right side shows the long-term cardio-metabolic outcomes (later adiposity, insulin resistance and higher blood pressure) with which rapid weight gain has been associated in observational studies. These associations are not established as causal; they may reflect confounding, or attained size rather than the tempo of gain, and meta-analyses have not found greater adiposity in childhood among infants born preterm or small for gestational age (main text, Sections 4.4.3 and 4.5). The balance is illustrative only: it does not imply that the outcomes on the right are likely, or that they can be weighed quantitatively against the short-term goals. The four steps summarize the practices set out in Section 7.3 of the main text; [Guideline] indicates that a step follows a current society guideline or position paper.
